# Cationic helicenes as selective G4 DNA binders and optical probes for cellular imaging†

**DOI:** 10.1039/d1sc04567a

**Published:** 2021-10-15

**Authors:** Peter A. Summers, Ajesh P. Thomas, Timothy Kench, Jean-Baptiste Vannier, Marina K. Kuimova, Ramon Vilar

**Affiliations:** Department of Chemistry, Molecular Sciences Research Hub, Imperial College London 82 Wood Lane, White City Campus W12 0BZ UK m.kuimova@imperial.ac.uk r.vilar@imperial.ac.uk +44 (0)20 7594 1967 +44 (0)20 7594 8558; Telomere Replication and Stability Group, Medical Research Council – London Institute of Medical Sciences London W12 0NN UK; Institute of Clinical Sciences, Faculty of Medicine, Imperial College London London W12 0NN UK

## Abstract

The important role that G-quadruplex DNA (G4 DNA) structures play in regulating biological processes is becoming widely recognised. These structures have also been proposed to be attractive drug targets. Therefore, there has been significant interest in developing small molecules that can selectively bind to G4 DNA over other topologies. In this paper we investigate the interaction between DNA and helical compounds (helicenes) based on a central carbocation trisubstituted with aromatic rings. We show that the non-planar structure of these helicenes results in a significantly reduced affinity for dsDNA when compared to their planar analogues, whilst maintaining a high affinity for G4 DNA. Additionally, the right- and left-handed enantiomers of one of these helicenes recognise the chiral DNA environments of G4 and dsDNA differently. We show that upon DNA binding the helicenes display a fluorescence switch-on effect, which we have successfully used for cellular imaging in live and fixed U2OS cells, staining mitochondria and the nucleus, respectively.

## Abstract 2

G-quadruplex DNA (G4 DNA) structures are selectively recognised by helical optical probes.

## Graphical abstract

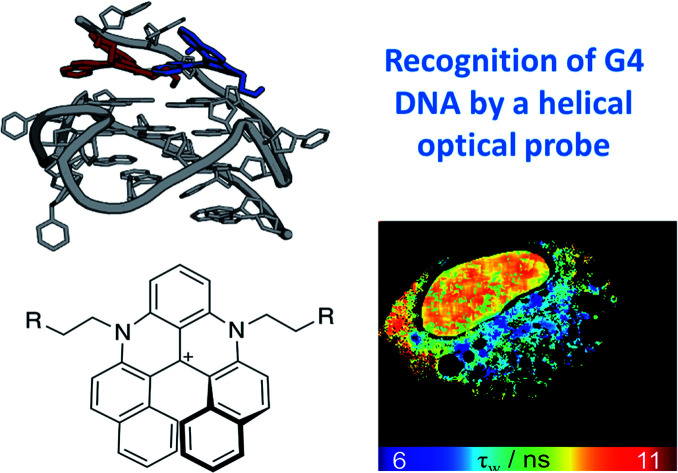

## Introduction

It is increasingly recognised that non-canonical DNA structures (*i.e.* non-duplex DNA) have important biological functions in living organisms.^1^ One such structure is the guanine-quadruplex (G4), a tetra-stranded helical assembly that forms in guanine-rich sequences of DNA. Bioinformatic studies as well as growing experimental evidence indicate that G4s are involved in a number of biological processes including telomere maintenance, replication and regulation of gene expression.^2,3^ Because of their proposed biological roles, G4s have been intensively studied as potential targets for the development of drugs, particularly for cancer.^4^

While G4s are thermodynamically stable and form readily *in vitro*, their presence in a cellular environment is proposed to be transient. This is due to the double stranded structure being the predominant topology in coiled DNA as well as the presence of dedicated helicases to unfold G4 structures.^5^ However, the formation and stability of G4s can be significantly enhanced in the presence of small molecules that preferentially bind to these tetra-stranded structures over other DNA topologies.^6–9^ Thus, G4 DNA binders can shift the duplex–quadruplex equilibrium and prevent helicases from resolving G4s.^5^ It has also been shown that small molecules can modify the interaction between G4s and proteins, particularly in the telomeres.^10^ This, together with the proposed biological roles of G4s, has prompted the development of a large number of small molecules designed to bind selectively to G4s over other topologies.^6,8,9,11^ Some of these molecules have been shown to trigger a number of biological responses in cells that are consistent with G4 stabilisation, while others have been successfully used as optical probes to visualise and detect G4 structures *in vitro* and, in a few cases, in live cells.^12–16^

Most G4 DNA binders are based on planar, polyaromatic molecules featuring positively charged substituents. As discussed extensively elsewhere, the planar core binds to the guanine tetrads *via* π–π interactions, while the substituents provide means to increase solubility and DNA affinity (*e.g.* with protonated amines), as well as selectivity for G4s over duplex DNA (dsDNA).^6,8,9,11^ While this strategy has yielded some very strong G4 DNA binders, alternative non-planar structural motifs have been explored with the aim of improving selectivity for a specific G4 topology, not only over duplex DNA, but also over other G4 topologies.^8^ One such strategy has been to exploit the explicit chiral environment formed in both duplex and G4 DNA, designing enantiomerically specific DNA binding molecules with stereoselectivity originating from octahedral metal-centres or from steric hindrance. For example, Thomas and co-workers studied the DNA binding properties of the di-ruthenium complex [{Ru(bipy)_2_}_2_(tpphz)]^4+^ where each of the two metal centres is in a chiral octahedral environment. They showed that the ΛΛ isomer has *ca.* 40 times higher affinity for human telomeric G4 DNA than the ΔΔ isomer.^17^ Another class of chiral G4 DNA binders are metallohelicenes, where two octahedral metal centres are bridged by three chelating ligands. For example, Qu and co-workers reported that one of the two enantiomers of a di-nickel metallohelice recognised *HTelo* G4 DNA with high affinity and selectivity over duplex DNA.^18–20^ Binding of related di-iron metallohelices to right and left-handed *HTelo* G4 DNA was recently investigated. The Δ, and Λ-enantiomers showed selectivity for right, and left-handed G4 DNA, respectively.^21^ Organic helical molecules such as foldamers have also been shown to bind G4s with high affinity and selectivity.^22^ It was proposed that the binding mode of these helical structures is different to that displayed by planar compounds – and likely to involve interactions with the backbone of G4 DNA. The M enantiomer of a cyclic helicene showed selectivity for B-DNA over the P isomer [*K*_d_(P)/*K*_d_(M) = 2.0], whereas the reverse trend was observed when binding Z-DNA [*K*_d_(P)/*K*_d_(M) = 0.3].^23^ Following on from this, a series of related chiral helicenes (with varying dihedral angles) showed enantioselective recognition of the M isomer to neighbouring G4s in the telomeric region.^24^

**Fig. 1:**
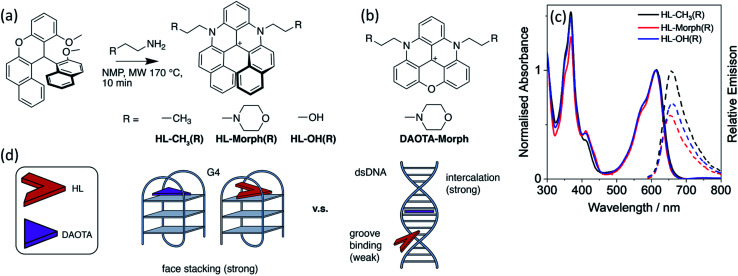
(a) Synthetic route and structures of **HL-CH3(R)**, **HL-Morph(R)**, **HL-OH(R)**, alongside (b), the structure of **DAOTA-Morph**. (c) Normalised absorbance (solid lines), and relative emission (dashed lines, *λ*_ex_ = 580 nm) of **HL-CH3(R)**, **HL-Morph(R)**, and **HL-OH(R)**, recorded in 10 mM lithium cacodylate buffer (pH 7.3) with 100 mM KCl. (d) Schematic drawing of the design strategy to lower the strength of binding for the helicenes (HL) to dsDNA whist maintaining strong binding to G4.

We have previously reported that a triangulenium, **DAOTA-Morph** (also known as **DAOTA-M2** – see Fig. 1), has good affinity for DNA and switches on its fluorescence upon binding.^12,15^ Its binding affinity towards different DNA topologies is *ca.* 2-fold higher for G4 than for duplex DNA (*K*_d_ values *ca.* 1.7 μM and 1.0 μM for duplex and G4 DNA structures respectively).^12,25^ Interestingly, the fluorescence lifetime of this probe is highly dependent on the DNA topology it binds to, which has allowed us to use **DAOTA-Morph** to probe the formation of G4 structures *via* fluorescence lifetime imaging microscopy (FLIM) in live cells.^12,15^

With the aim of improving further the selectivity of this type of probe for specific G4 structures over other DNA topologies, herein we present studies with the helical analogues of trianguleniums, namely cationic helicenes. This type of compound, previously reported by Lacour and co-workers,^26^ can often be resolved into their stereoisomers and they are emissive over a wide range of wavelengths, depending on bridging atoms and substituents. These helical trianguleniums derivatives have been used as dyes for cellular imaging and shown to accumulate in mitochondria of live cells, and in one report, they were shown to bind to duplex DNA.^27^ Herein we report the synthesis of the new racemic (R) helicences **HL-OH(R)** and **HL-Morph(R)** (the latter being a direct analogue of the planar **DAOTA-Morph** compound which has been successfully used as a lifetime-based optical probe for G4s^12,15^) and their DNA binding properties, which are compared with those of the previously reported helicene **HL-CH3(R)**.^26,28–32^ Further, the racemic mixture of **HL-OH(R)** was partially resolved into the corresponding M and P stereoisomers. We show that these compounds have high selectivity for G4 DNA over duplex DNA; this is particularly the case for the **HL-OH(M)** stereoisomer. We also show that in live cells **HL-Morph(R)** is cell permeable and localises in mitochondria, whereas in fixed cells nuclear staining of DNA is possible, as confirmed using FLIM.

## Results and discussion

### Design and synthesis of helicenes as DNA binders

As stated above, the planar aromatic molecule **DAOTA-Morph** [Fig. 1(b)] binds to dsDNA and G4 DNA with similar affinities.^12^ We aimed to reduce the probe's affinity for dsDNA, whilst maintaining strong G4 binding, thus resulting in improved selectivity for G4 over dsDNA. To achieve this, we designed and synthesised a cationic helicene molecule **HL-Morph(R)** [Fig. 1(a)] in which steric hindrance induced by extension of the aromatic system, causes distortion away from a planar structure. This nonplanar helical topology should in principle be prevented from intercalating into adjacent bases in duplex DNA, favouring a weak groove binding arrangement [Fig. 1(d)]. On the other hand, the helical molecule still has the appropriate structural features to bind to G4 DNA *via* end-stacking. We also synthesised **HL-CH3(R)** as a control, and **HL-OH(R)** which we were able to partially separate into the enantiomers **HL-OH(P)** and **HL-OH(M)** as confirmed by CD spectroscopy and chiral HPLC (see below).

These helicenes were synthesised using a multi-step synthetic strategy based on that previously reported by Lacour and co-workers for similar helical molecules (Fig. 1 and S1†).^26^ The key intermediate 11-methoxy-12-(2-methoxynaphthalen-1-yl)-12*H*-benzo[*a*]xanthen-12-ylium tetrafluoroborate [**1** in Fig. S1†] was prepared in bulk.^26^ This was heated with the corresponding amine in NMP to form the cationic racemic mixtures of the corresponding diaza-helicenes, namely **HL-CH3(R)**, **HL-Morph(R)** and **HL-OH(R)**. The crude compounds were purified by flash chromatography and characterised by ^1^H NMR and ^13^C NMR spectroscopy, and ESI-MS (Fig. S2–S7†).

### **HL-CH3(R)**, **HL-Morph(R)** and **HL-OH(R)** binding affinities to ctDNA and *c-Myc*

**Fig. 2:**
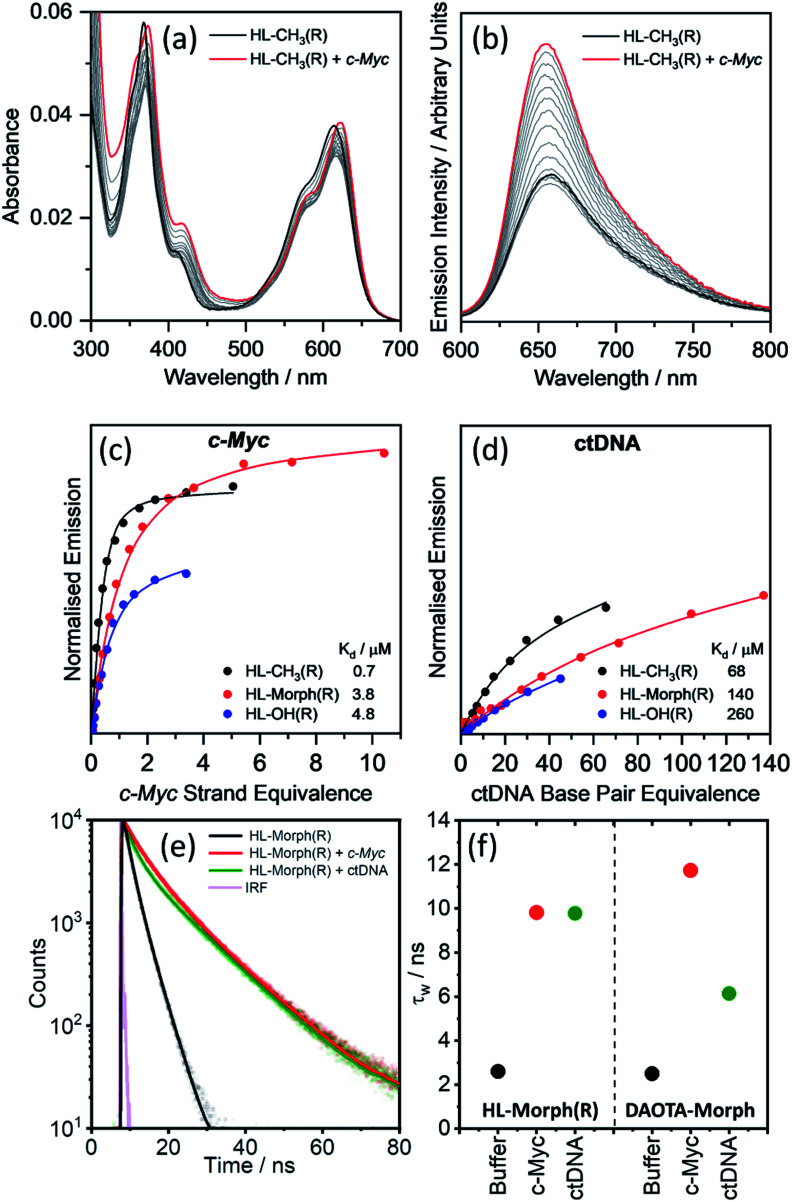
Binding affinities of **HL-CH3(R)**, **HL-Morph(R)**, and **HL-OH(R)** to *c-Myc* and ctDNA. (a) Absorption and (b) emission spectra of **HL-CH3(R)** (4 μM) during addition of *c-Myc* G4 DNA (0–5 strand equivalence). Difference in integrated emission (*λ*_ex_ = 580 nm, *λ*_em_ = 600–700 nm) during titration with (c) *c-Myc* and (d) ctDNA. Integrated intensities are normalised against the absorption at the excitation wavelength. The solid line is the best fit of a simple binding model (see Methods section and Fig. S10† for binding models used) to solve for *K*_H_ and *k*_Δ*G*H_. For all titration data recorded for **HL-CH3(R)**, **HL-Morph(R)**, and **HL-OH(R)** see Fig. S8 and S9(e) and (f)† fluorescence lifetime analysis of **HL-Morph(R)** in buffered aqueous solution and bound to different oligonucleotide topologies. (e) Time resolved fluorescence decays of **HL-Morph(R)** (2.5 μM, black trace) and following the subsequent additions of G4 (*c-Myc*, 10 strand equivalence, red dots), and dsDNA (ctDNA, 140 base pair equivalence, green dots). Solid lines are bi-exponential fits of the decay traces. (f) Variation in average lifetime (*τ*_w_) of the bi-exponential fits in (e). Results are presented alongside the corresponding *τ*_w_ values recorded for **DAOTA-Morph**, adapted from ref. 12. All experiments performed in 10 mM lithium cacodylate buffer (pH 7.3) with 100 mM KCl.

We first set out to investigate the binding affinities of **HL-CH3(R)**, **HL-Morph(R)**, and **HL-OH(R)** to dsDNA (ctDNA) and G4 DNA (*c-Myc*). As indicated above, **DAOTA-Morph**, the planar aromatic analogue of **HL-Morph(R)**, binds to ctDNA and G4 DNA with similar binding affinities.^12^ By distorting the aromatic structure of **DAOTA-Morph** to make **HL-Morph(R)**, we aimed to maintain strong π–π stacking to the G4 quartet, whilst reducing affinity for dsDNA. These helicenes are fluorescent and their emission intensity in aqueous buffered media is switched-on upon DNA binding. Therefore, we were able to study the DNA binding affinity of **HL-CH3(R)**, **HL-Morph(R)** and **HL-OH(R)** through titrations with ctDNA and *c-Myc* G4 DNA (see Fig. 2 and S8, S9†). The UV/visible spectra undergo a red-shift in absorption maximum during addition of DNA [Fig. 2(a)], as the fluorescence intensity increases [Fig. 2(b)].

Fitting the fluorescence titrations' data [see Methods section and Fig. S10† for binding models used], the dissociation constants for *c-Myc* [*K*_d_ = 0.7–4.8 μM, Fig. 2(c)] are comparable to those displayed by **DAOTA-Morph** (*K*_d_ = 0.8 μM for *myc2345*). However, those for ctDNA [*K*_d_ = 68–260 μM, Fig. 2(d)] show that the interaction of these helicenes with dsDNA is at least 50 times weaker than that displayed by **DAOTA-Morph** [*K*_d_ = 1.3 μM]. G4 binding was confirmed using a fluorescent intercalator displacement (FID) assay,^33^ with all three helicene complexes showing a similar ability to displace TO from *c-Myc* (Fig. S9†).

We next investigated the fluorescence lifetime (*τ*_w_, a concentration independent parameter) of the helicenes upon binding to DNA. When free in aqueous buffer at pH 7.3, the helicenes' *τ*_w_ ranges between 2.6 and 5.1 ns (Fig. S12†) while, upon DNA binding, a significant increase in lifetime to between 8 and 12 ns is observed (see Fig. 2 and S12†). Although the helicenes' fluorescence lifetime cannot be used to discriminate between different DNA topologies (unlike **DAOTA-Morph**^12,15^), the significant increase of *τ*_w_ when bound to DNA was useful for cellular imaging studies in order to confirm that the dyes are bound to DNA in cellular organelles (see below).

### Binding modes of **HL Morph(R)** and **DAOTA-Morph** to dsDNA

Given that **HL-Morph(R)** is *ca.* 100 times weaker binder towards ctDNA than **DAOTA-Morph** (Table 1), we investigated if this difference could be a result of a different binding mode. Structural perturbations of DNA induced by dye binding can be monitored using CD spectroscopy and used to investigate the dye binding mode. The characteristic CD spectrum of ctDNA (in its B form) shows an increase in the band at 277 nm upon **DAOTA-Morph** binding [Fig. S13(a) and (b)†]. Similar spectral changes have been observed following the intercalation of planar aromatic dyes into dsDNA, tentatively assigned to the unwinding of the helical DNA structure to accommodate the intercalated dye.^34,35^ Conversely, when the same number of **HL-Morph(R)** molecules per base pair are bound to ctDNA, a small decrease in band intensity at 277 nm is observed [Fig. S13(a) and (b)†]. This minor change in the CD signal is characteristic of groove binding which results in minimal disruption to the double helical structure.^36,37^ We next studied the accessibility of ctDNA bound **DAOTA-Morph** and **HL-Morph(R)** to fluorescence quenching by iodide. If intercalated, the proximity of base pairs above and below the dye should protect against quenching, whereas this shielding will be less for groove binding dyes which are still exposed to the solvent environment. Once bound, **DAOTA-Morph** is almost completely protected from quenching by iodide [*K*_SV_ = 21.8 (free) and 3.1 (bound) M^−1^], whereas for **HL-Morph(R)** the quenching is in fact enhanced [*K*_SV_ = 36.1 (free) and 48.7 (bound) M^−1^, Fig. S13(c)†]. This seemingly unusual enhancement in quenching when bound to dsDNA has been observed for other positively charged, weak groove binding dyes,^36,38^ which was assigned to weakened electrostatic **HL-Morph(R)**-dsDNA interaction through increasing ionic strength of the solvent upon the addition of *K*I. As a result, **HL-Morph(R)** is released into solution allowing more efficient quenching. Molecular docking studies provided further evidence that **DAOTA-Morph** intercalates into dsDNA whilst **HL-Morph(R)** interacts *via* groove binding. These studies showed that **HL-Morph(R)** is too wide to fit into an intercalation site (Fig. S14†), whereas **DAOTA-Morph** fits well. Taken together, this evidence indicates that distortion of the aromatic surface in **HL-Morph(R)** away from a planar structure did indeed result in a change in binding mode, which favours a weak groove binding mode over intercalation into dsDNA. This in turn accounts for the high selectivity of the helicenes for G4 DNA (over ctDNA) confirming our original hypothesis.

**Table 1:** *K*_d_ values (in μM), fluorescence switch-on, and selectivity values for **HL-CH3(R)**, **HL-Morph(R)** and **HL-OH(R)**, binding to *c-Myc* and ctDNA. All experiments in 10 mM lithium cacodylate buffer (pH 7.3) with 100 mM KCl

	*c-Myc*		*ctDNA*		*c-Myc* : *ctDNA*
	*K*_d_	Switch-on	*K*_d_	Switch-on	Selectivity^a^
**HL-CH3(R)**	0.7	1.8	68	1.8	97
**HL-Morph(R)**	3.8	2.7	140	2.6	37
**HL-OH(R)**	4.7	1.9	260	1.9	55
**DAOTA-Morph** b	0.8	*ca.* 5	1.3	*ca.* 2.5	1.6

a Selectivity values calculated as *K*_d_ ctDNA/*K*_d_*c-Myc*.

b Values for **DAOTA-Morph** binding to *myc2345* and ctDNA from ref. 12.

### **HL-OH(P)** and **HL-OH(M)** binding affinities to *c-Myc*, *BCL2*, *HTG4* and ctDNA

**Fig. 3:**
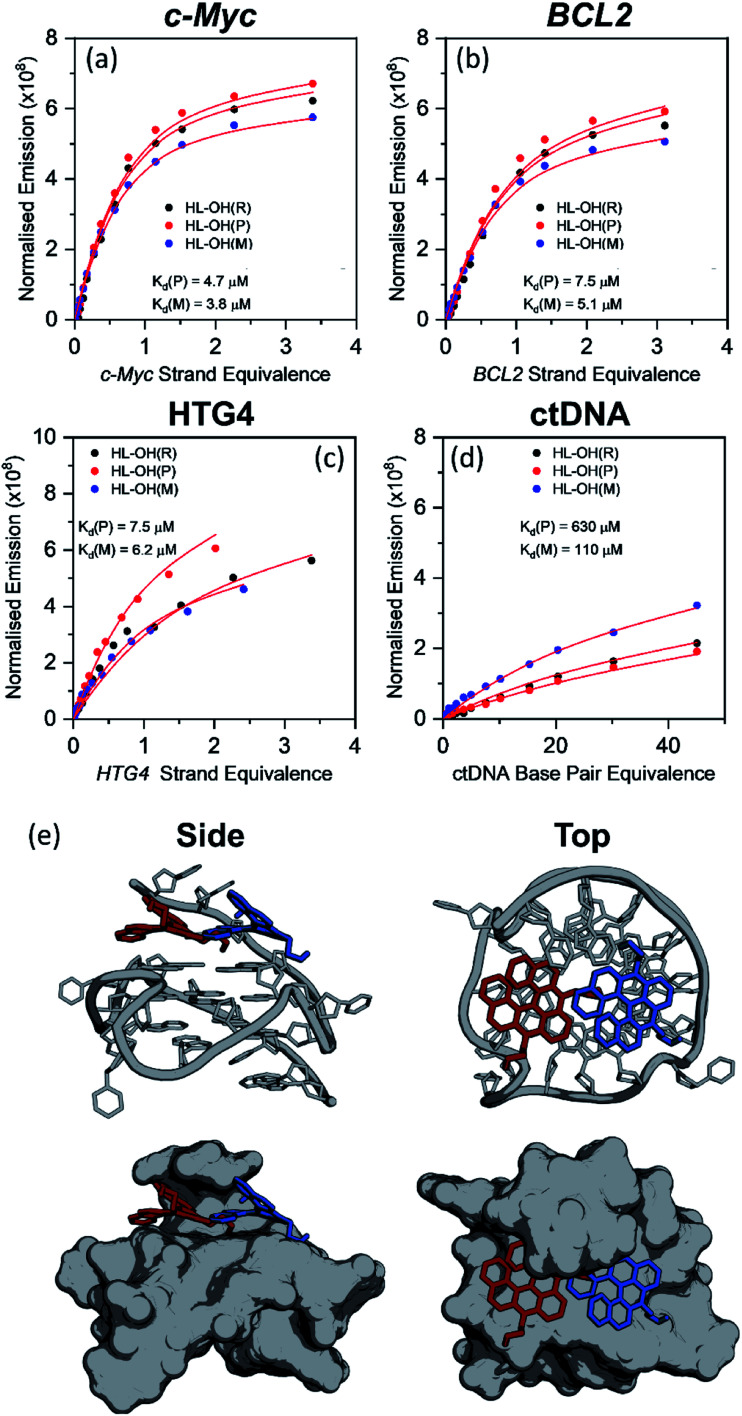
Plots showing the competitive binding of **HL-OH(R)** (6 μM, 0% ee), **HL-OH(P)** (6 μM, 32% ee), and **HL-OH(M)** (6 μM, 96% ee) to (a) *c-Myc*, (b) *BCL2* (c) *HTG4* and (d) ctDNA. Data points are the difference in integrated emission during titration, normalised against the absorption at the excitation wavelength. The solid red line is a simultaneous best fit of all three titrations to a competitive binding model (Fig. S9†), to solve for *K*_P_, *K*_M_, *k*_Δ*G*_P__ and *k*_Δ*G*_M__. All experiments were performed in 10 mM lithium cacodylate buffer (pH 7.3) with 100 mM KCl. For all titration data see Fig. S13–S16.† (e) Lowest energy orientation following molecular docking of **HL-OH(P)** (red), and **HL-OH(M)** (blue) to *c-Myc*. The 5′ overhang creates a chiral pocket for the helicene to bind in.

As discussed in the previous section, **HL-CH3(R)**, **HL-Morph(R)**, and **HL-OH(R)** show high selectivity for G4 over dsDNA (Table 1), so we therefore investigated if the individual enantiomers (P and M) would show different DNA binding properties. We were able to partially resolve **HL-OH(R)** (0% ee) into **HL-OH(P)** (32% ee) and **HL-OH(M)** (96% ee) as evidenced by chiral HPLC [Fig. S22b†]; attempts to resolve **HL-Morph(R)** were unsuccessful. The binding affinities of the partially-resolved sample of **HL-OH(P)** and **HL-OH(M)** towards *c-Myc*, *BCL2*, and *HTG4* quadruplexes, as well as ctDNA, were determined (Fig. 3). To improve the accuracy of fitting, titrations of **HL-OH(R)**, **HL-OH(P)** and **HL-OH(M)** were fitted simultaneously to a competitive binding model (see Methods section and Fig. S10† for details) to solve for *K*_d_(P) and *K*_d_(M).

**HL-OH(M)** consistently binds more strongly to both G4 and dsDNA than **HL-OH(P)**. For *c-Myc* [*K*_d_ = 4.7 and 3.8 μM for **HL-OH(P)** and **HL-OH(M)**, respectively, Fig. 3 and S15†] and *HTG4* [*K*_d_ = 7.5 and 6.2 μM for **HL-OH(P)** and **HL-OH(M)**, respectively, Fig. 3 and S17†] this difference is small, presumably because the open face of these quadruplex structures presents a modestly chiral environment for binding.^39^ Using the published NMR-structure of *c-Myc* incorporating a G4-binder,^39^ we performed docking studies on the ligand free structure [Fig. 3(e)]. This confirmed the independent biding preferences for both **HL-OH(P)** and **HL-OH(M)** into a chiral pocket caused by the 5′ overhang of the G4. We next investigated binding of **HL-OH(P)** and **HL-OH(M)** to a mixed parallel/antiparallel quadruplex structure that forms in the promoter region of the *BCL2* gene, as it contains loop regions that interact with both G-tetrad faces, potentially forming an increased chiral environment for binding.^40^ Indeed, the difference in binding between **HL-OH(P)** and **HL-OH(M)** [*K*_d_ = 7.5 μM and 5.1 μM, respectively, Fig. 3 and S14†] is slightly increased when interacting with *BCL2*, as compared to *c-Myc* and *HTG4*.

A much bigger difference in affinity between the two isomers was observed when binding to ctDNA [Fig. 3 and S18†]. **HL-OH(P)** [*K*_d_ = 630 μM, Fig. 3(c)] shows a reduced interaction with ctDNA compared to **HL-OH(M)** [*K*_d_ = 110 μM, Fig. 3(c)], with selectivity for *c-Myc* over ctDNA of 134 and 29, respectively. As binding to *BCL2* is weaker, the selectivity over ctDNA is decreased for both **HL-OH(P)** and **HL-OH(M)** (84 and 22, respectively) compared to *c-Myc*.

Given the large difference in binding to ctDNA between the P and M isomers, we investigated if this difference could be observed by CD spectroscopy when **HL-Morph(R)** is bound to a large excess of ctDNA. Racemic **HL-Morph(R)** shows no bands in the CD spectrum, however, once added to a large excess of ctDNA, negative CD bands at 314, 374 and 472 nm develop [Fig. S19(a)†]. The appearance of a CD signal in a region outside of any ctDNA absorption implies the enrichment of one isomer bound to ctDNA compared to one isomer free in solution. Based on the measured spectra of free **HL-OH(M)**, and **HL-OH(M)** bound to ctDNA, we calculated CD spectra that would be expected if either the M or the P isomers had the stronger association constant [Fig. S19(b)†]. The spectrum expected for the M isomer strongly bound to ctDNA, closely matches the experimental spectrum, confirming the preference of the M isomer in binding to ctDNA.

### **HL-Morph(R)** staining in live and fixed U2OS cells

**Fig. 4:**
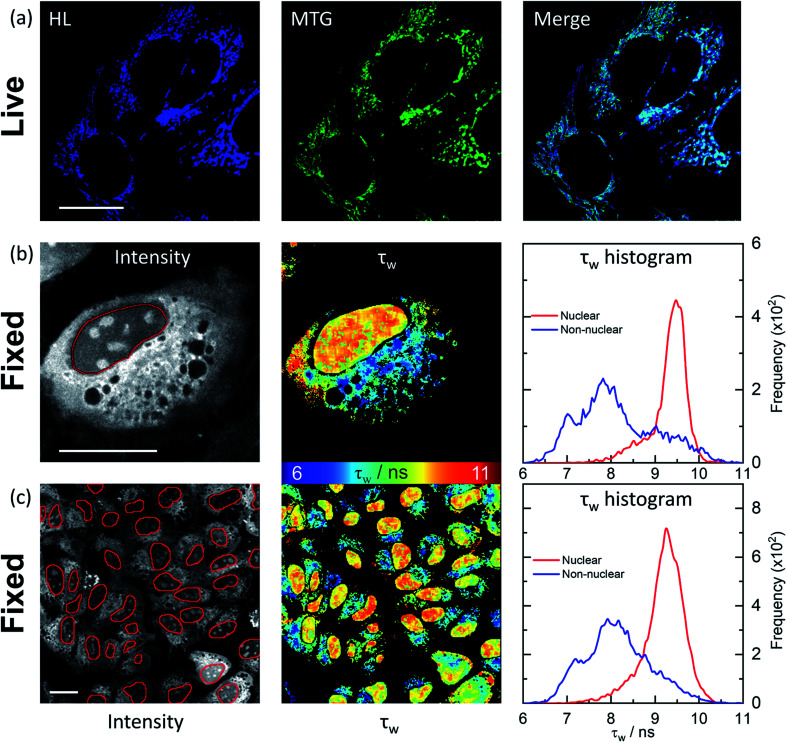
Confocal and FLIM images of **HL-Morph(R)** incubated with live and fixed U2OS cells. (a) Confocal images (1024 × 1024 resolution) of helicene fluorescence (left: HL, 10 μM, 24 h, *λ*_ex_ = 633 nm, *λ*_em_ = 650–790 nm) and co-incubated with Mito-Tracker Green (middle: MTG, 50 nM, 45 min, *λ*_ex_ = 488 nm, *λ*_em_ = 500–600 nm). Right: image merge confirms co-localisation. (b) and (c): FLIM analysis of fixed U2OS cells stained with **HL-Morph(R)** (20 μM, 0.5 h, *λ*_ex_ = 640 nm, *λ*_em_ = 650–790 nm). Left: fluorescence intensity images recorded at (b) 512 × 512 (c) and 256 × 256 resolution, red lines represent the nuclear segmentation used for the FLIM analysis. Middle: corresponding FLIM maps. Right: histogram of fluorescence lifetime distribution. For *χ*^2^ maps see Fig. S21.† Scale bars: 20 μm.

We next investigated how **HL-Morph(R)** stains live and fixed U2OS cells. We chose **HL-Morph(R)** due to low toxicity to live cells (Fig. S20†), synthetic ease and the ability to compare against our previous results with structurally related lifetime-based G4 probe, **DAOTA-Morph**.^12^ At the concentrations used in our live cell experiments (10 μM, 24 h), the cytotoxicity of **HL-Morph(R)** is negligible. Similarly to other previously reported helicene compounds,^27*a*^**HL-Morph(R)** accumulates in mitochondria of live cells, confirmed by co-localisation with mitotracker green [MTG, Fig. 4(a)]. Again, this contrasts with **DAOTA-Morph**, which localises predominantly in the nucleus of live U2OS cells.^12^

In fixed U2OS cells, **HL-Morph(R)** passes the nuclear membrane, revealing both nuclear and non-nuclear staining [Fig. 4(b) and (c)]. Recording FLIM maps of these fixed cells, intensity weighted average lifetimes recorded within the nucleus are represented by a histogram with a peak maximum at *ca.* 9.5 ns, consistent with *in vitro* experiments for **HL-Morph(R)** bound to DNA [9.8 ns regardless of topology, Fig. 2(f)]. We note that significantly higher fluorescence intensity can be observed in the nucleoli [Fig. 4(b), left panel], although the concentration and topology-independent lifetime measurement does not reveal any reason for this.

## Conclusions

The vast majority of G4 DNA binders reported in the literature are based on planar polyaromatic systems. While planar molecules have high affinities to G4 DNA due to efficient π–π end-stacking, they also tend to intercalate efficiently into duplex DNA. Herein we have demonstrated that by breaking the planarity of polyaromatic systems, it is possible to generate much more selective G4 binders. More specifically, we have shown that the fluorescent helicene compounds, **HL-CH3(R)**, **HL-Morph(R)**, and **HL-OH(R)** have high selectively for G4 over duplex DNA, and further selectivity can be introduced through partial chiral resolution of **HL-OH(R)** into **HL-OH(P)** and **HL-OH(M)**. The distorted core of the helicene compound reduces affinity for dsDNA compared to the planar analogue **DAOTA-Morph**, whist maintaining strong affinity for G4 DNA [selectivity = 134 for **HL-OH(P)**]. We also show that **HL-OH(P)** and **HL-OH(M)** bind differently to G4 and dsDNA topologies, with **HL-OH(M)** consistently displaying a higher affinity. We have also used the increased fluorescence intensity and lifetime of **HL-Morph(R)** upon DNA binding to enable cellular imaging studies. In live U2OS cells this helicene accumulates in the mitochondria, whereas in fixed cells, **HL-Morph(R)** passes the nuclear membrane and binds to DNA, as confirmed using fluorescence lifetime imaging microscopy, FLIM.

## Methods

### General synthetic procedures

All chemicals were purchased from commercial sources and used as received, unless stated otherwise. ^1^H and ^13^C NMR spectra were recorded using either a 400 or 500 MHz Bruker Avance Ultrashield NMR spectrometer at 296 K. Spectra were referenced internally by using the residual solvent (^1^H *δ* = 3.34 and ^13^C *δ* = 49.86 for CD_3_OD-*d*_4_ relative to SiMe_4_). ESI-MS spectra were recorded by Dr L. Haigh (Imperial College London) on a Bruker Daltronics Esquire 3000 spectrometer. **HL-CH3(R)** was synthesised and characterised according to published procedures.^26^

### Synthesis of **HL-Morph(R)**

Compound **1** (0.1 g, 0.198 mmol) and 4-(2-aminoethyl)morpholine (0.644 g, 4.96 mmol) were mixed with anhydrous 1-methyl-2-pyrrolidinone (NMP) (1 mL) under an argon atmosphere in a microwave (MW) tube. This reaction mixture was stirred in a MW synthesizer for 10 min at 170 °C then allowed to cool down to room temperature. CH_2_Cl_2_ (5 mL) was added to the reaction mixture and was washed with a 1 M aqueous solution of HBF_4_ (2 × 2 mL). The organic phase was separated and dried over anhydrous Na_2_SO_4_ and evaporated under vacuum. The resulting crude material was purified using flash chromatography to yield **HL-Morph(R)** (0.015 g, 11%). ^1^H NMR (400 MHz, CD_3_OD): *δ* 8.43 (d, ^3^*J* = 12 Hz, 2H), 8.34 (t, ^3^*J* = 8 Hz, 1H), 8.21 (d, ^3^*J* = 12 Hz, 2H), 7.98 (d, ^3^*J* = 8 Hz, 2H), 7.92 (d, ^3^*J* = 8 Hz, 2H), 7.36 (t, ^3^*J* = 8 Hz, 2H), 7.10 (d, ^3^*J* = 8 Hz 2H), 6.77 (t, ^3^*J* = 8 Hz, 2H), 5.23–5.17 (m, ^3^*J* = 8 Hz, 2H), 5.16–4.98 (m, ^3^*J* = 8 Hz, 2H), 3.70–3.55 (m, 8H), 3.07 (t, ^3^*J* = 8 Hz 4H), 2.65 (t, ^3^*J* = 8 Hz, 8H). ^13^C NMR (100 MHz, CD_3_OD): *δ* 144.8, 143.8, 140.6, 140.5, 137.0, 131.7, 131.1, 130.5, 129.5, 129.2, 124.7, 118.4, 117.9, 109.5, 68.8, 57.0, 55.9. ESI-MS-*m*/*z* calculated for C_39_H_39_N_4_O_2_^+^ = 595.31 a.m.u.; found = 595.46 a.m.u.

### Synthesis of **HL-OH(R)**

Compound **1** (0.1 g, 0.198 mmol) and freshly distilled 2-amino ethanol (0.352 g, 4.96 mmol) was mixed with anhydrous 1-methyl-2-pyrrolidinone (NMP) (1 mL) under an argon atmosphere in a MW tube. This reaction mixture was stirred in a MW synthesiser for 10 min at 170 °C then allowed to cool down to room temperature. CH_2_Cl_2_ (5 mL) was added to the reaction mixture and was washed with a 1 M aqueous solution of HBF_4_ (2 × 2 mL). The organic phase was separated, dried over anhydrous Na_2_SO_4_, and evaporated under vacuum. The resulting crude material was purified using flash chromatography to yield the desired helicene **HL-OH(R)** (0.018 g, 16%). ^1^H NMR (500 MHz, CD_3_OD): *δ* 8.40 (d, ^3^*J* = 10 Hz, 2H), 8.28 (t, ^3^*J* = 5 Hz, 3H), 8.03 (d, ^3^*J* = 5 Hz, 2H), 7.89 (d, ^3^*J* = 10 Hz, 2H), 7.32 (t, ^3^*J* = 7.5 Hz, 2H), 7.15 (d, ^3^*J* = 5 Hz, 2H), 6.77 (t, ^3^*J* = 7.5 Hz, 2H), 5.20–5.16 (m, 2H), 5.02–4.97 (m, 2H), 4.34–4.28 (m, 4H). ^13^C NMR (125 MHz, CD_3_OD): *δ* 144.61, 140.13, 139.50, 135.97, 130.96, 130.58, 129.57, 128.66, 128.40, 124.17, 117.56, 117.41, 109.00, 60.32, 52.62, ESI-MS-*m*/*z* calculated for C_31_H_25_N_2_O_2_^+^ = 457.19 a.m.u.; found = 457.19 a.m.u.

### Partial enantiomeric resolution of **HL-OH(R)** into **HL-OH(P)** and **HL-OH(M)**

Partial enantiomeric resolution was achieved by adopting a reported protocol, using the enantiomeric phosphorous complex, [Me_2_NH_2_][Λ-BINPHAT].^26^**HL-OH(R)** (18 mg, 0.033 mmol) and [Me_2_NH_2_][Λ-BINPHAT] (33.80 mg, 0.039 mmol) were dissolved in a CH_2_Cl_2_/acetone (1/1 2 mL) mixture in a vial. The solution was stirred for 30 min at RT after which time the solvent was removed under reduced pressure. The solid obtained was dissolved in 3 mL acetone and kept overnight at 0 °C. The solid deposited at the bottom of the vial was separated from the mother liquor. The solvent of the latter was removed in a rotary evaporator to yield a second solid. CH_2_Cl_2_ was added to each solid fraction (the original precipitate and the solid obtained after evaporation) followed by HPF_6_ (9 mg) and 0.6 mL KPF_6_ (0.1 M solution in water) and stirred for 1 h. The organic layers were separated and purified using flash chromatography.

To confirm the enantiomeric resolution of **HL-OH(R)**, samples were analysed by chiral HPLC as follows: a 100 μM stock solution of the corresponding compound (*i.e.***HL-OH(R)**, **HL-OH(P)** or **HL-OH(M)**) was prepared in MeOH. 1.5 equivalents of NaBH_4_ were added, causing an immediate colour change from blue to colourless – due to reaction of the helicene carbocation as described previously.^26^ The solution was left for 6 h to allow for full NaBH_4_ hydrolysis. Next, the corresponding solution was diluted to 50 : 50 hexane : MeOH before injection onto a Chiralpak AD-H 250 × 4.6 mm column. An isocratic 60 : 40 hexane : isopropanol gradient was run and the peaks corresponding to each stereoisomer integrated to give an ee of 96% for **HL-OH(M)** and 32% for **HL-OH(P)** (see Fig. S22(b)† for chromatograms). The two enantiomers were analysed by CD spectroscopy and the spectra are shown in Fig. S22(a).†

### General methods for spectroscopic and biophysical studies

Stock solutions of **HL-CH3(R)**, **HL-Morph(R)**, **HL-OH(R)**, **HL-OH(P)** and **HL-OH(M)** were prepared in DMSO and the concentration determined in CH_2_Cl_2_ using the molar extinction coefficient 16 596 M^−1^ cm^−1^ published for **HL-CH3(R)** at 616 nm.^26^ The enantiomeric excess (ee) of **HL-OH(P)** (ee = 32%) and **HL-OH(M)** (ee = 96%) were calculated using chiral HPLC [Fig. S22(b)†] as described above, and confirmed by CD spectroscopy in CH_2_Cl_2_ [Fig. S22(a)†] using the published Δ*ε* values for **HL-CH3(P)** (107 Δ*ε*, 96% ee) and **HL-CH3(M)** (−87.3 Δ*ε*, 92% ee).^26^*c-Myc* (5′-TGAGGGTGGGTAGGGTGGGTAA-3′) *BCL2* (5′-GGGCGCGGGAGGAATTGGGCGGG-3′) and *HTG4* (5′-AGGGTTAGGGTTAGGGTTAGGG -3′) were purchased from Eurogentec. A two-nucleotide mutated form of the *BCL2* gene was used, namely *BCL2Mid*, as it favours formation of a well-defined single structure.^40^ This modified structure is referend to as *BCL2* throughout this manuscript. The oligonucleotides were dissolved in 10 mM lithium cacodylate buffer at pH 7.3. KCl was added to a final concentration of 100 mM, then annealed at 95 °C for 10 min. Calf thymus DNA (ctDNA, Sigma) were dissolved in the same cacodylate buffer, and KCl added to a final concentration of 100 mM. All oligonucleotide concentrations were determined in salt free buffer (before any annealing) using the molar extinction coefficients 228 700 M^−1^ cm^−1^ (strand for *c-Myc*), 227 300 M^−1^ cm^−1^ (strand for *BCL2*), 228 500 M^−1^ cm^−1^ (strand for *HTG4*) and 13 200 M^−1^ cm^−1^ (base pair for CT-DNA). Concentrations of G4 and dsDNA are expressed as per strand, and per base pair, respectively.

Fluorescence spectra were recorded using a Fluoromax-4 spectrofluorimeter (Jobin-Yvon; Horiba). Absorbance spectra were recorded using an 8453 UV-Visible Spectroscopy System (Agilent). Circular Dichroism (CD) spectra were recorded using either a J-810 (JASCO) or V100 (Chirascan) CD spectrophotometer.

### Time-correlated single photon counting (TCSPC)

Time-resolved fluorescence decays were obtained using an IBH 5000F (Jobin Ybon, Horiba) time-correlated single photon counting (TCSPC) device equipped with a 635 nm NanoLED as an excitation source (pulse width <200 ps, HORIBA) with a 100 ns time window and 4096 time bins. Decays were detected at *λ*_em_ = 655 nm (±4 nm) after passing through a 645 nm long pass filter to remove any scattered excitation pulse. Decays were accumulated to 10 000 counts at the peak of fluorescence decay. A neutral density filter was used for the instrument response function (IRF) measurements using a *Ludox* solution, detecting the emission at the excitation wavelength. Decay traces were fitted by iterative reconvolution to the equation *I*(*t*) = *I*_0_(*α*_1_e^−*t*/*τ*_1_^ + *α*_2_e^−*t*/*τ*_2_^) where *α*_1_ and *α*_2_ are variables normalised to unity. The intensity-weighted average lifetime (*τ*_w_) was calculated using the equation:1
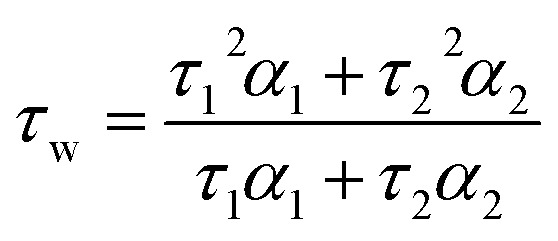


A prompt shift was included in the fitting to take into account differences in the emission wavelength between the IRF and measured decay. The goodness of fit was judged by consideration of the deviations from the model *via* a weighted residuals plot.

### Oligonucleotide titrations

The helicene under study (2–6 μM) was dissolved in 10 mM lithium cacodylate buffer (pH 7.3) supplemented with 100 mM KCl and the UV/visible/fluorescence/CD spectra and/or TCSPC lifetime recorded (where applicable). Increasing amounts of the oligonucleotide under study were added, maintaining a constant concentration of the helicene compound. After each addition, the mixture was left to equilibrate for >30 s (a time determined to be sufficient for equilibrium to occur) before the corresponding photophysical measurements were recorded. Fluorescence spectra (*λ*_ex_ = 580 nm, *λ*_ex_ = 590–850 nm) were integrated between 600 and 800 nm and the integrated intensity normalised against the absorption at the excitation wavelength. Titrations are plotted as Δ*F*, the difference between the normalised emission, and the normalised emission of the free helicene (first point of the titration). For **HL-OH(M)** in aqueous buffer, broadening of the absorbance spectral bands can be observed, consistent with compound aggregation. In this case, Δ*F* was calculated as the difference between the normalised emission, and the normalised emission from the first addition of oligonucleotide (second point of the titration).

Titration curves were fitted to either a simple binding (which assumes the 2 stereoisomers are equivalent)^41,42^ or competitive binding (which assumes independent binding for each stereoisomer)^43,44^ models using a modified form of the MatLab script reported previously (Fig. S10†).^41,42^ Based on the titration data, a binding stoichiometry of two compounds to one G-quadruplex and two compounds per five base pairs for ctDNA was used allowing for direct comparison with **DAOTA-Morph**.^12^ No co-operativity between binding sites and no oligonucleotide fluorescence response was assumed. Titrations of racemic mixtures of **HL-CH3(R)**, **HL-Moph(R)** and **HL-OH(R)** were fitted independently to the simple binding model to solve for the association constant (*K*_H_) and the fluorescence change on binding (*k*_Δ*G*_H__). Titrations with **HL-OH(R)** (0% ee), **HL-OH(P)** (32% ee) and **HL-OH(M)** (96% ee) were fitted simultaneously to the competitive binding model solve for *K*_P_, *K*_M_, *k*_Δ*G*_P__ and *k*_Δ*G*_M__. In the case of ctDNA, a complete titration was not possible due to a low binding affinity, which resulted in a less accurate fit. To account for this, the values of *k*_Δ*G*_P__ and *k*_Δ*G*_H__ were fixed from the results of competitive binding to *c-Myc*. Reported *K*_d_ values are the reciprocal of the association constant. The fluorescence ‘switch-on’ values for **HL-CH3(R)**, **HL-Morph(R)**, and **HL-OH(R)** were calculated as *F*/*F*_0_, where *F* is calculated from the asymptotic value of the binding fit, and *F*_0_ is the initial point on the binding curve, before the addition of oligonucleotide. N.B., this value includes correction for absorption at the excitation wavelength. The selectivities of G4 over dsDNA was calculated as ratios of *K*_d_ values (Table 1).

### Thiazole orange fluorescence indicator displacement assay (TO-FID)

Experiments were carried out using a BMG CLARIOstar® Microplate reader with Greiner Bio-One half volume (100 μl well) plates using a method adapted from the literature.^33^ Fluorescence titrations (*λ*_ex_ = 475 nm, *λ*_ex_ = 520 nm) were carried out using *c-Myc* G4 DNA (100 mM KCl, 10 mM Tris-HCl, pH 7.3). The final concentrations in the plate were 1 μM G4 DNA, 2 μM thiazole orange (TO) and 0–20 μM helicene (0, 0.94, 1.25, 1.88, 2.50, 3.75, 5.0, 7.5, 10, 15, 20). Sample preparation was carried out by first preparing double concentration stocks of helicene (40 and 30 μM) and a DNA/TO mixture (2 μM and 4 μM respectively). Helicene concentrations were prepared using serial dilutions at 50 μL per well, which was followed by the addition of 50 μL of the DNA/TO mixture and gently shaken for 5 minutes. Percentage displacement curves were calculated from the measured fluorescence intensity (*F*), using: displacement  = 100 − [(*F*/*F*_0_) × 100], where *F*_0_ is TO fluorescence from the probe bound to *c-Myc* without added helicene. Displacements were fitted to a Hill function which was used to calculate the DC_50_.

### General cell culture

Human bone osteosarcoma epithelial cells (U2OS, from ATCC) were grown in high glucose Dulbecco's Modified Eagle Medium (DMEM) containing 10% fetal bovine serum (FBS) at 37 °C with 5% CO_2_ in humidified air.

### Cytotoxicity of **HL-Morph(R)** and **HL-CH3(R)**

Cytotoxicity of **HL-Morph(R)** and **HL-CH3(R)** in U2OS cell lines were investigated using the MTS assay. The cells were equally distributed (5 × 10^3^ cells per well) in a 96 well plate in a DMEM medium containing 10% FBS and incubated for 12 h under standard condition. The culture media was removed, and fresh media added with compounds at required concentrations (20, 10, 5, 2.5, 1.25 and 0.625 μM) in triplicate. Wells were maintained without compound (only cells) and without cells (only culture medium) as 100% and 0% viability controls, respectively. After 24 h incubation the cells were treated with MTS/PMS solution and incubated for another 4 h before taking an absorption reading using a plate reader.

### Confocal imaging

1024 × 1024 resolution fluorescence images were collected using an inverted confocal laser scanning microscope (Leica SP5 II). MitoTracker Green (MTG) emission (500–600 nm) was collected following one-photon excitation from an internal microscope laser at 488 nm, and helicene emission collected at (650–790 nm) following excitation from an internal microscope laser at 633 nm.

### Fixed cell experiments

Cells were seeded on chambered coverglass (1.5 × 10^4^ cells, 250 μL, 0.8 cm^2^) for 48 h. Cells were washed (*x*3) in ice cold PBS before incubation in ice cold paraformaldehyde (PFA, 4% in PBS) solution for 10 min, and a further wash (*x*3) with ice cold PBS. Fixed cells were further treated with **HL-Morph(R)** (20 μM, 0.5 h, 21 °C) in PBS before being left under PBS. Cells were left under PBS for imaging to limit the effect of refractive index of the fixation medium on the florescence lifetime.^45^

### Fluorescence lifetime imaging microscopy (FLIM)

FLIM was performed through time-correlated single-photon counting (TCSPC), using an inverted confocal laser scanning microscope (Leica SP5 II) and a SPC-830 single-photon counting card (Becker & Hickl GmbH). A pulsed diode laser (Becker & Hickl GmbH, 640 nm, 20 MHz) was used as the excitation source, with a PMC-100-1 photomultiplier tube (Hamamatsu) detector. Fluorescence emission (650–790 nm) was collected through an Airy 1 pinhole for an acquisition time sufficient to obtain signal strength suitable for decay fitting. For all live cell imaging, cells were mounted (on chambered coverglass slides) in the microscope stage, heated by a thermostat (Lauda GmbH, E200) to 37 (±0.5)°C, and kept under an atmosphere of 5% CO_2_ in air. A 100× (oil, NA = 1.4) objective was used to collect images at either 256 × 256 or 512 × 512 pixel resolution, as stated in the text. The IRF used for deconvolution was recorded using reflection of the excitation beam from a glass cover slide.

Lifetime data were fitted using the FLIMfit software tool developed at Imperial College London (v5.1.1, Sean Warren, Imperial College London) to a bi-exponential function, and the intensity-weighted lifetime (*τ*_w_) calculated using eqn (1). 5 × 5 and 9 × 9 square binning was used to increase signal strength for images recorded at 256 × 256 and 512 × 512 resolution, respectively. A scatter parameter was added to the decay fitting to account for scattered excitation light. Before fitting, a mask was applied to the images to analyse individual cell nuclei staining, or extra-nuclear staining. A threshold was applied to the average of each nucleus to require a minimum of 175 at the peak of the decay and a goodness-of-fit measured by *χ*^2^ of less than 2.

### Molecular docking

Molecular docking was performed using AutodocVina.^46^ Ligand structures of isolated compounds were minimised in Gaussian using Density Functional Theory (DFT), the B3LYP functional, and a 6-31G(d,p) basis. Compounds were docked into the lowest energy form of *c-Myc* (PDB ID:5W77),^39^ or dsDNA (PDB ID:1Z3F),^47^ already stripped of their bound ligands. A grid box encompassing the entire quadruplex was used for blind docking. The lowest energy solution was used and the docked structures were visualised using PyMol v2.3.4.

## Data availability

The essential spectroscopic characterisation and analytical data is included in the ESI.† Additional data is available from the corresponding authors upon reasonable request.

## Author contributions

J. B. V., M. K. K. and R. V. conceptualised the study and acquired funding. P. A. S., A. P. T., J. B. V., M. K. K. and R. V. designed the methodology and performed the investigation. A. P. T. synthesised and characterised the compounds. P. A. S. and A. P. T. performed the formal analysis. T. K. performed the TO displacement experiments and enantiomeric resolution by HPLC. P. A. S., A. P. T., M. K. K. and R. V. co-wrote and edited the paper.

## Conflicts of interest

There are no conflicts to declare.

## Supplementary Material

SC-012-D1SC04567A-s001
