# Supplementary material for: Cationic helicenes as selective G4 DNA binders and optical probes for cellular imaging
Source: Chem Sci. 2021 Oct 15;12(43):14624–34. doi: 10.1039/d1sc04567a (PMC8580066; doi:10.1039/d1sc04567a)
Supplement: SC-012-D1SC04567A-s001 [file SC-012-D1SC04567A-s001.pdf]

Electronic Supplementary Material (ESI) for Chemical Science.
This journal is © The Royal Society of Chemistry 2021

Cationic helicenes as selective G4 DNA binders and optical probes for cellular imaging

Peter A. Summers,[1#] Ajesh P. Thomas,[1#] Timothy Kench, Jean-Baptiste Vannier,[2,3] Marina K. Kuimova*,[1] Ramon Vilar*[1]

Supplementary Information

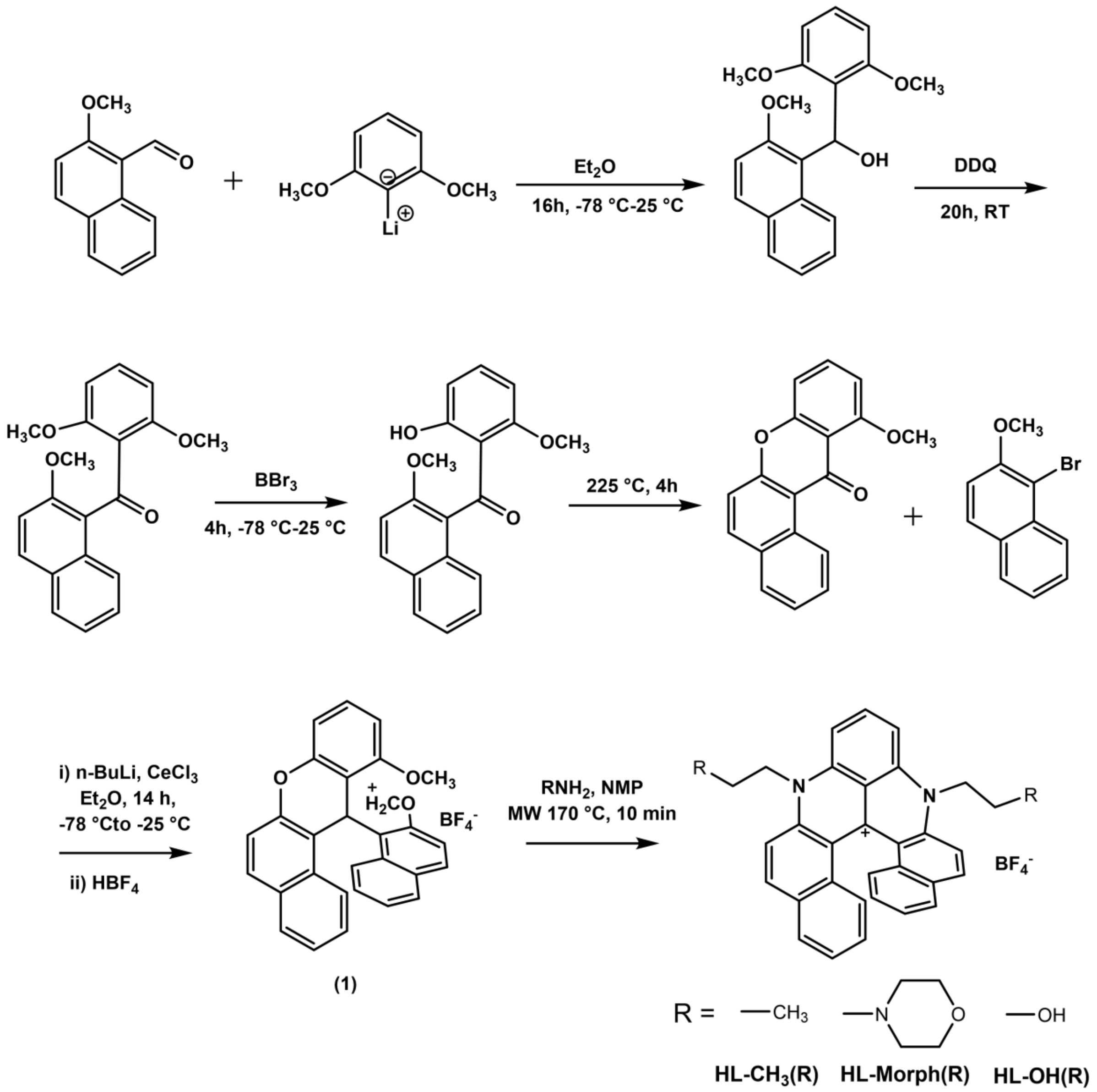

Figure S1. Synthetic pathway to **HL-CH$_3$(R)**, **HL-Morph(R)**, and **HL-OH(R)**.

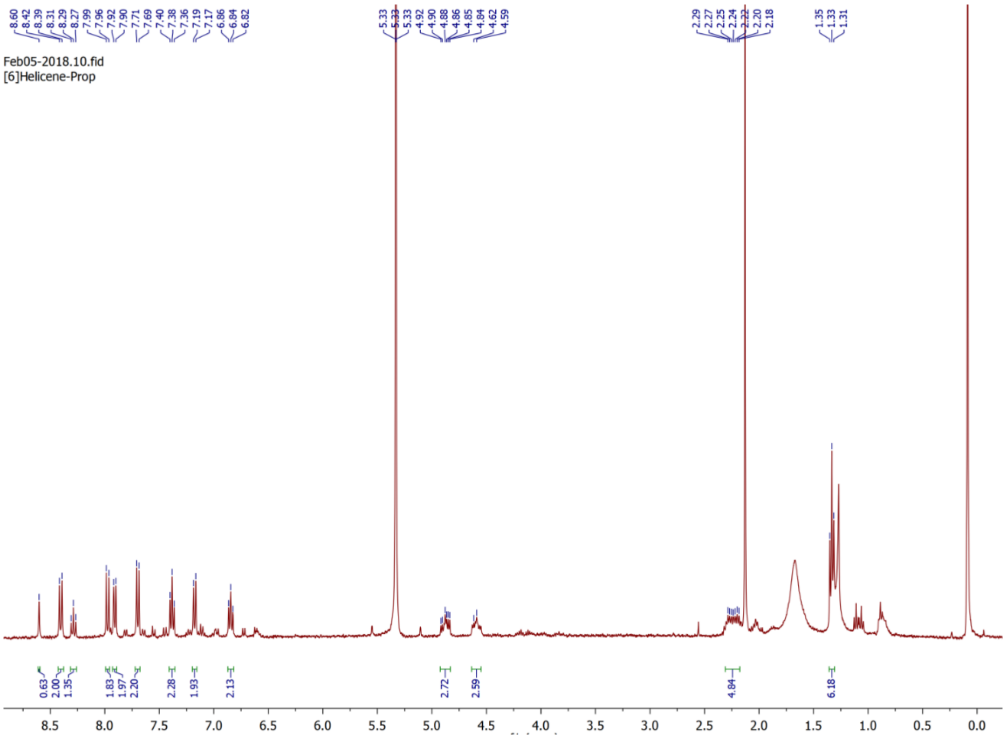

Figure S2. ^{1}H NMR spectrum of **HL-CH$_3$(R)** in CD_3OD.

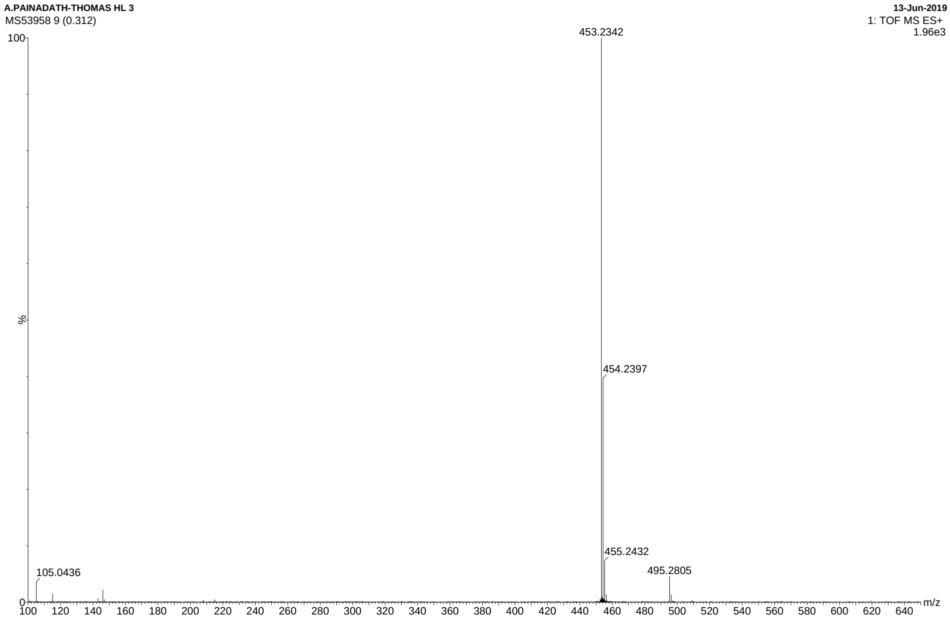

Figure S3. ESI-MS spectrum of **HL-CH$_3$(R)**.

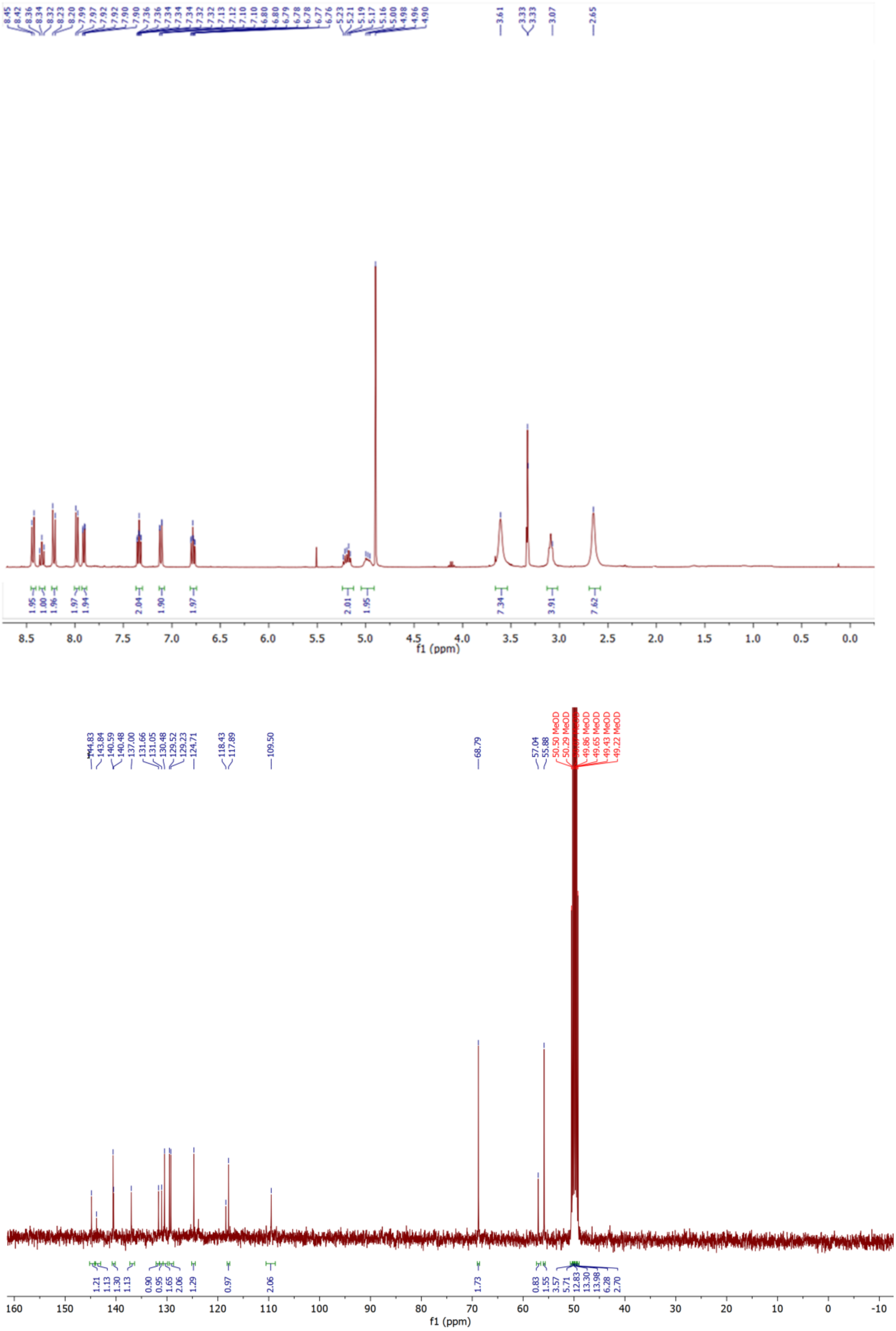

Figure S4. ^{1}H NMR (top) and ^{13}C NMR (bottom) spectra of **HL-Morph(R)** in CD_3OD.

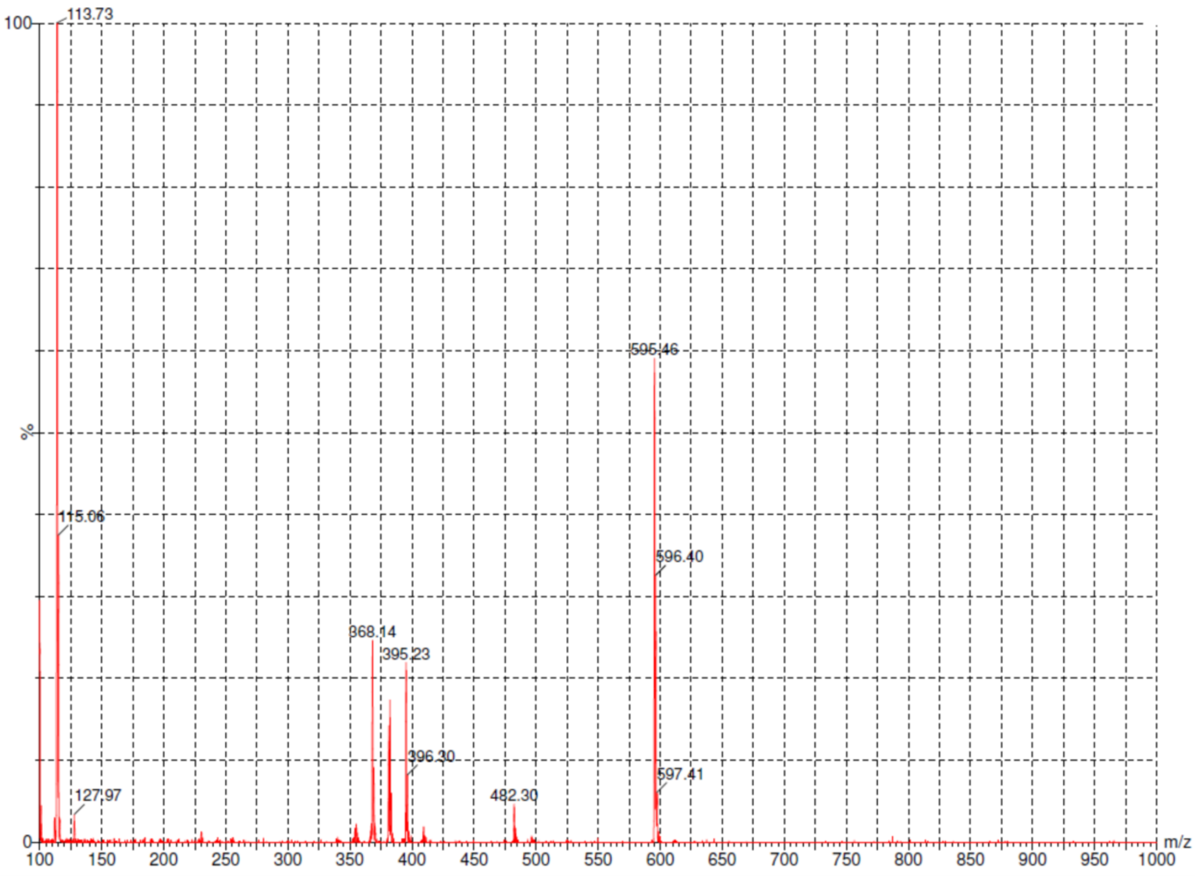

Figure S5. ESI-MS spectrum of **HL-Morph(R)**.

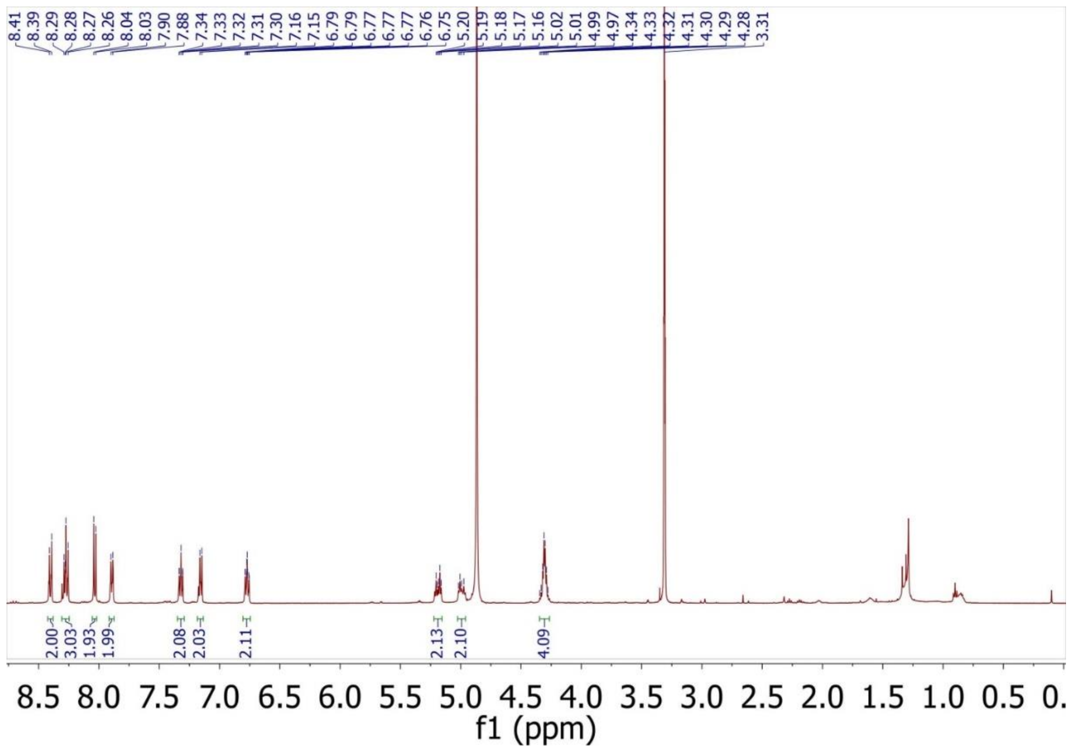

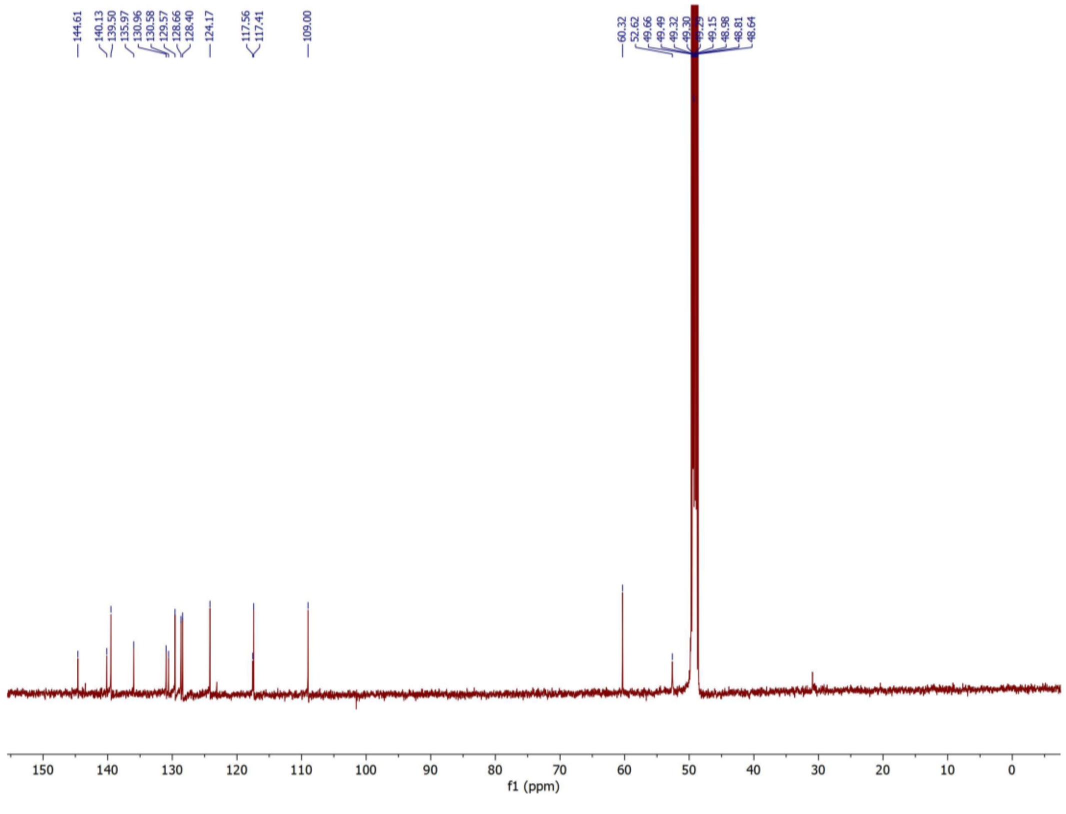

Figure S6. ^{1}H NMR (top) and ^{13}C NMR (bottom) spectra of **HL-OH(R)** in CD_3OD.

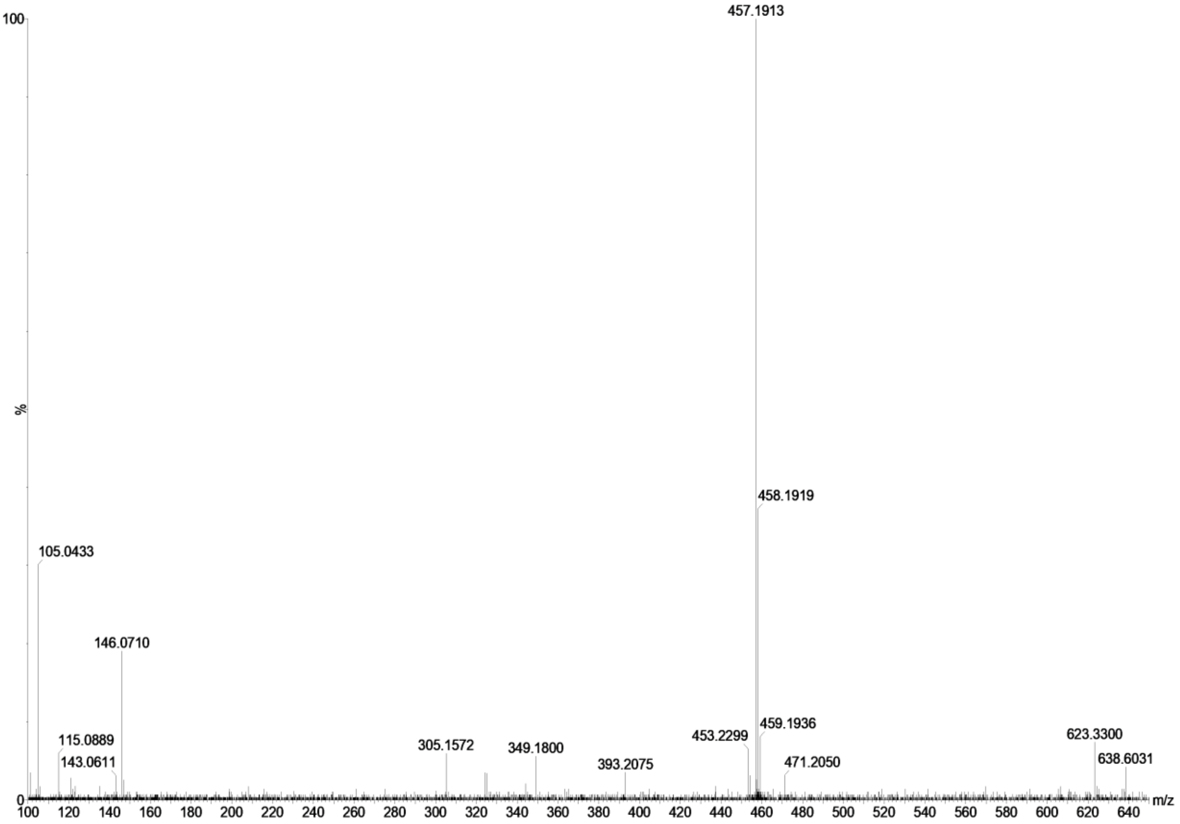

Figure S7. ESI-MS spectrum of **HL-OH(R)**.

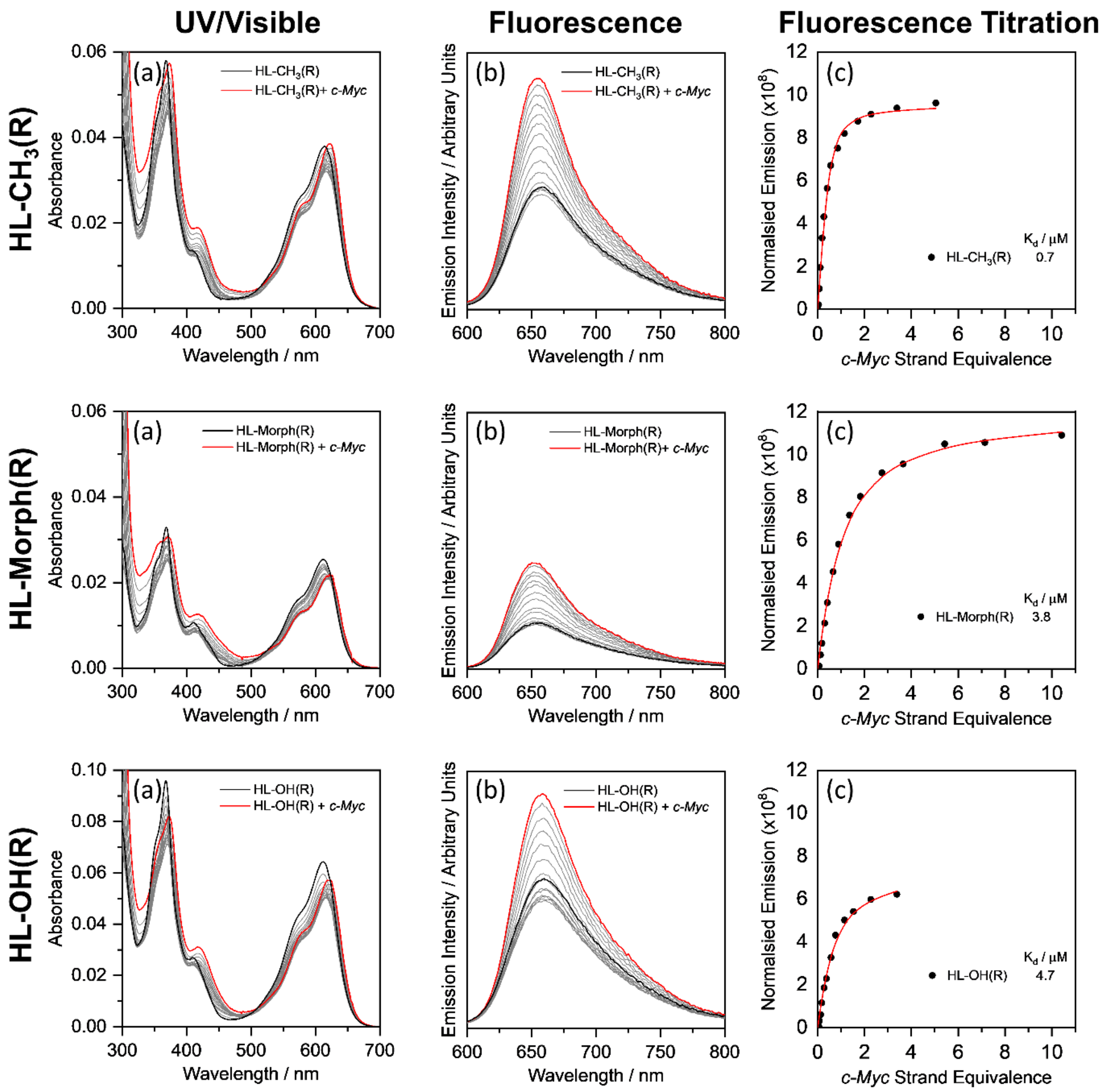

Figure S8. Titration spectra and simple binding affinities of **HL-CH₃(R)** (4 µM), **HL-Morph(R)** (2.5 µM), and **HL-OH(R)** (6 µM) to *c-Myc* G4 DNA. (a) Absorption spectra and (b) Fluorescence spectra (λ_{ex} = 580 nm, λ_{ex} = 600-700 nm) during addition of *c-Myc*. (c) Difference in Integrated emission during titration. Integrated intensities are normalised against absorption at the excitation wavelength. The solid red line is a best fit of a simple binding model. All experiments in 10 mM lithium cacodylate buffer (pH 7.3) with 100 mM KCl.

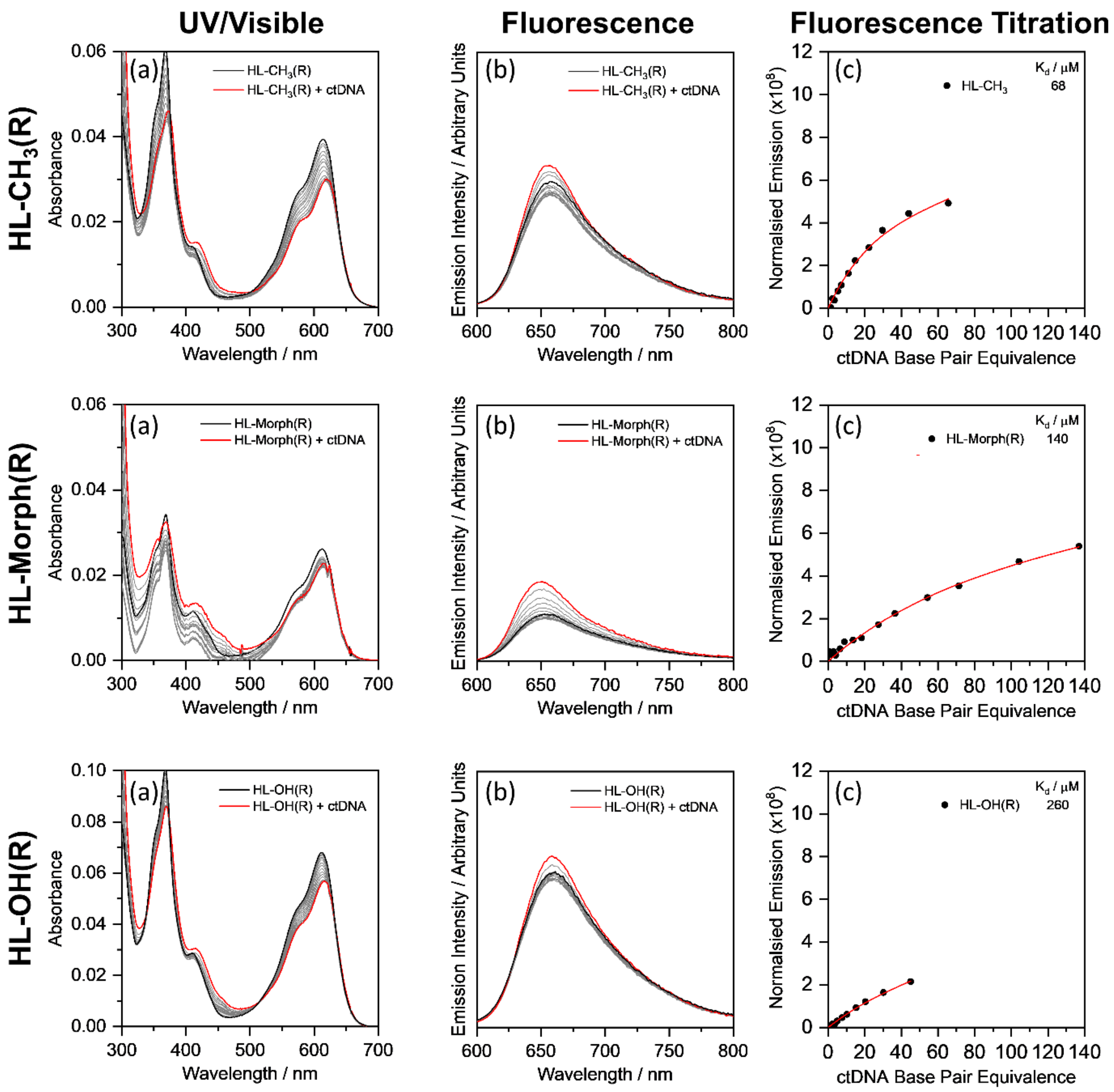

Figure S9. Titration spectra and simple binding affinities of **HL-CH$_3$(R)** (4 µM), **HL-Morph(R)** (2.5 µM), and **HL-OH(R)** (6 µM) to ctDNA DNA. (a) Absorption spectra and (b) Fluorescence spectra (λ_{ex} = 580 nm, λ_{ex} = 600-700 nm) during addition of ctDNA DNA. (c) Difference in Integrated emission during titration. Integrated intensities are normalised against absorption at the excitation wavelength. The solid red line is a best fit of a simple binding model. All experiments in 10 mM lithium cacodylate buffer (pH 7.3) with 100 mM KCl.

Simple

Equilibria

$$G + H \underset{}{\overset{K_H}{\rightleftharpoons}} GH \quad K_H = \frac{[GH]}{[G][H]}$$

$$[G]_0 = [G] + [GH]$$
$$[H]_0 = [H] + [GH]$$

Guest Concentration

$$a[G]^2 + b[G] + c = 0$$
$$a = 1$$
$$b = -[G]_0 + [H]_0 + K_H{}^{-1}$$
$$c = -[G]_0 \times K_H{}^{-1}$$

Fluorescence Change

$$\Delta F = k_{\Delta GH}[GH]$$

Competitive

Equilibria

$$G + P \overset{K_P}{\rightleftharpoons} GP \quad K_P = \frac{[GP]}{[G][P]}$$

$$G + M \overset{K_M}{\rightleftharpoons} GM \quad K_M = \frac{[GM]}{[G][M]}$$

$$[G]_0 = [G] + [GP] + [GM]$$
$$[P]_0 = [P] + [GP]$$
$$[M]_0 = [M] + [GM]$$

Guest Concentration

$$a[G]^3 + b[G]^2 + c[G] + d = 0$$
$$a = K_P K_M$$
$$b = KP + K_M + K_P K_M\ ([M]_0 + [P]_0 - [G]_0)$$
$$c = 1 + K_P\ ([P]_0 - [G]_0) + K_M\ ([M]_0 - [G]_0)$$
$$d = -[G_0]$$

Fluorescence Change

$$\Delta F = \frac{k_{\Delta GP} K_P [G][P]_0}{1 + K_P[G]} + \frac{k_{\Delta GM} K_M\ [G][M]_0}{1 + K_M[G]}$$

Figure S10. Equilibrium equations used to fit titration data to either simple[1, 2] or competative[3, 4] binding models using a modified form of the MatLab script reported previously.[1, 2] G = binding sites in the oligonucleotides (two compounds to one G-quadruplex and two compounds per five base pairs for ctDNA) as per reference 5 for **DAOTA-Morph**), H = helicene (racemic mixtures), P = **HL-OH(P)**, M = **HL-OH(M)**. $k_{\Delta GH}$ $k_{\Delta GP}$ $k_{\Delta GM}$ = difference in emission intensity between bound and free H, P and M, respectively. Fluorescence response of G assumed to be 0. No cooperativity between binding sites assumed. Titrations with **HL-OH(R)** (0% ee), **HL-OH(P)** (32% ee) and **HL-OH(M)** (96% ee) were fitted simultaneously to solve for K_P, K_M, $k_{\Delta GP}$ and $k_{\Delta GM}$.

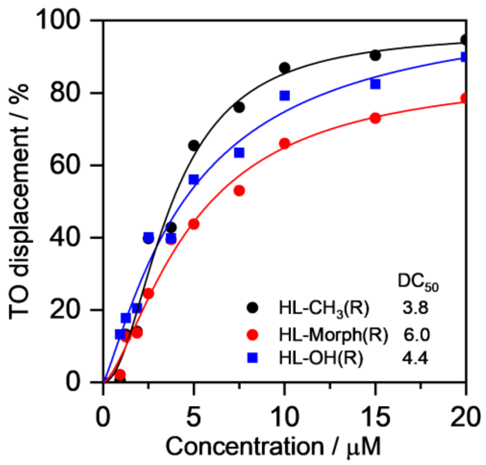

Figure S11. TO G4-FID curves for **HL-CH$_3$(R)**, **HL-Morph(R)**, and **HL-OH(R)** (λ_{ex} = 475 nm, λ_{em} = 520 nm). Solid lines are Hill function fits of the displacement curves. All experiments in 10 mM TRIS-HCl buffer (pH 7.3) with 100 mM KCl.

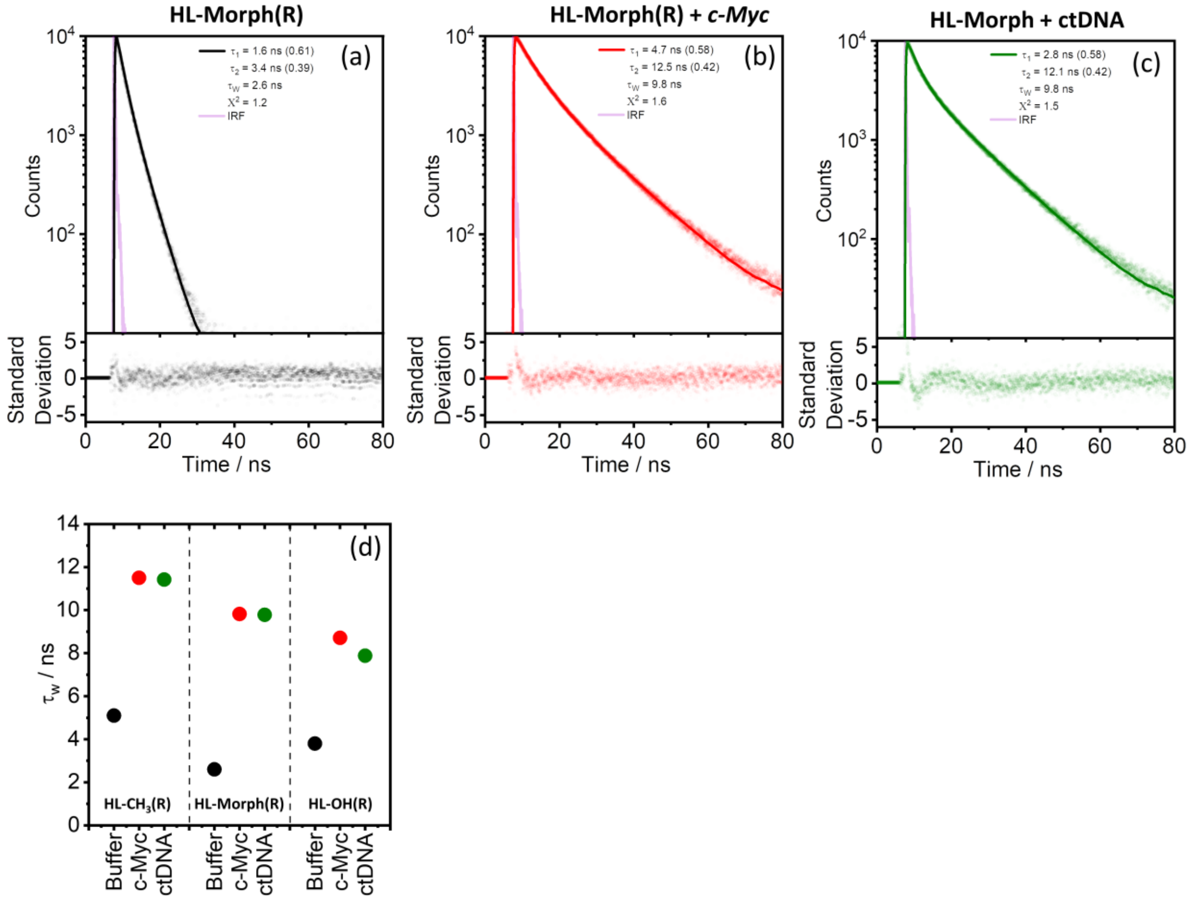

Figure S12. (a) Time resolved fluorescence decays of **HL-Morph(R)** in buffered aqueous solution and bound to different oligonucleotide topologies. G4 (*c-Myc*, 10 strand equivalence, red dots), dsDNA (ctDNA, 140 base pair equivalence, green dots) and RNA (tRNA, 140 base equivalence, blue dots). Solid lines are bi-exponential fits of the decay traces. (b) Variation in average lifetime (τ_w) of **HL-CH$_3$(R)**, **HL-Morph(R)**, and **HL-OH(R)** in buffered aqueous solution and when bound to *c-Myc*, and dsDNA. All experiments in 10 mM TRIS-HCl buffer (pH 7.3) with 100 mM KCl.

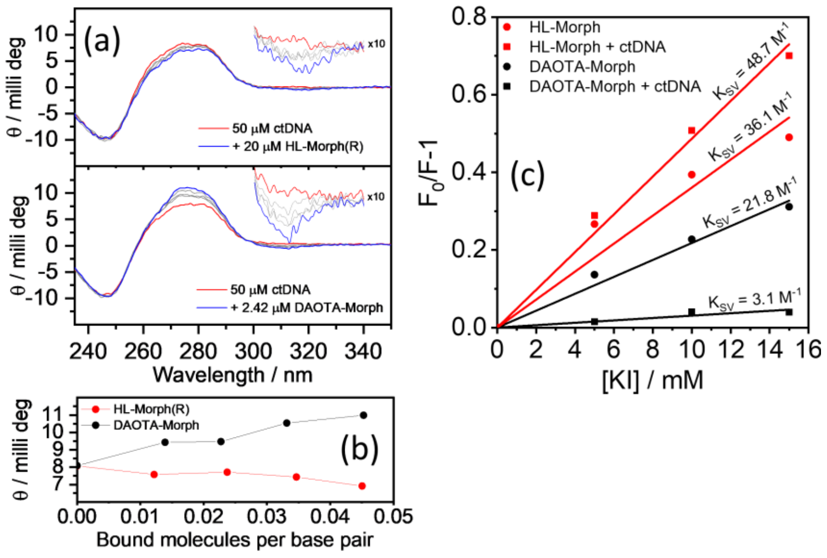

Figure S13. (a) CD spectra of ctDNA (50 μM) during addition (0 to 0.045 bound molecules per base pair) of **HL-Morph(R)** (top) and **DAOTA-Morph** (bottom). Inset: 10x expansion in the y-axis for the range 300 – 340 nm. In both titrations, the amount of dye added was calculated to keep the ratio of bound molecules per dsDNA base pair constant. (b) Change in θ at 277 nm during the titration shown in (a). (c) Quenching by KI of **HL-Morph(R)** and **DAOTA-Morph** (both at 2 μM) when 100% free dye or bound to dsDNA (818 and 11 μM, respectively). In both experiments with dsDNA, the amount of bound dye was constant at 70%. Calculations of bound dye concentrations were performed using the simple binding equation in Figure S10, and the K_d values for **HL-Morph(R)** (140 μM, Table 1) and **DAOTA-Morph** (1.3 μM, reference [5]).

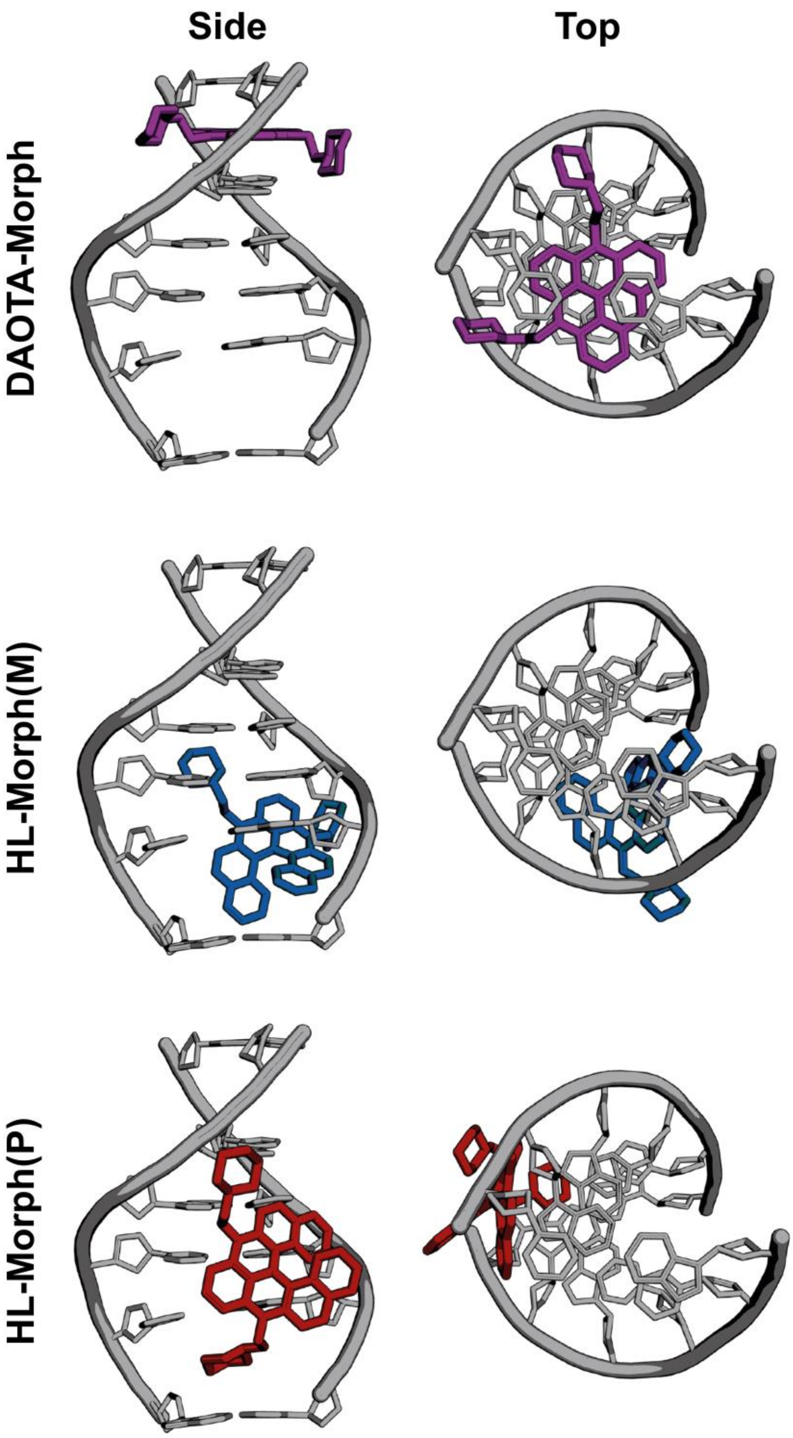

Figure S14. Lowest energy orientation following molecular docking of **HL-Morph(P)** (red), **HL-Morph(M)** (blue) and **DAOTA-Morph** (magenta) to dsDNA containing an intercalation binding site. Planar **DAOTA-Morph** can intercalate into the binding site, whereas **HL Morph(P)** (red), and **HL Morph(M)** does not fit.

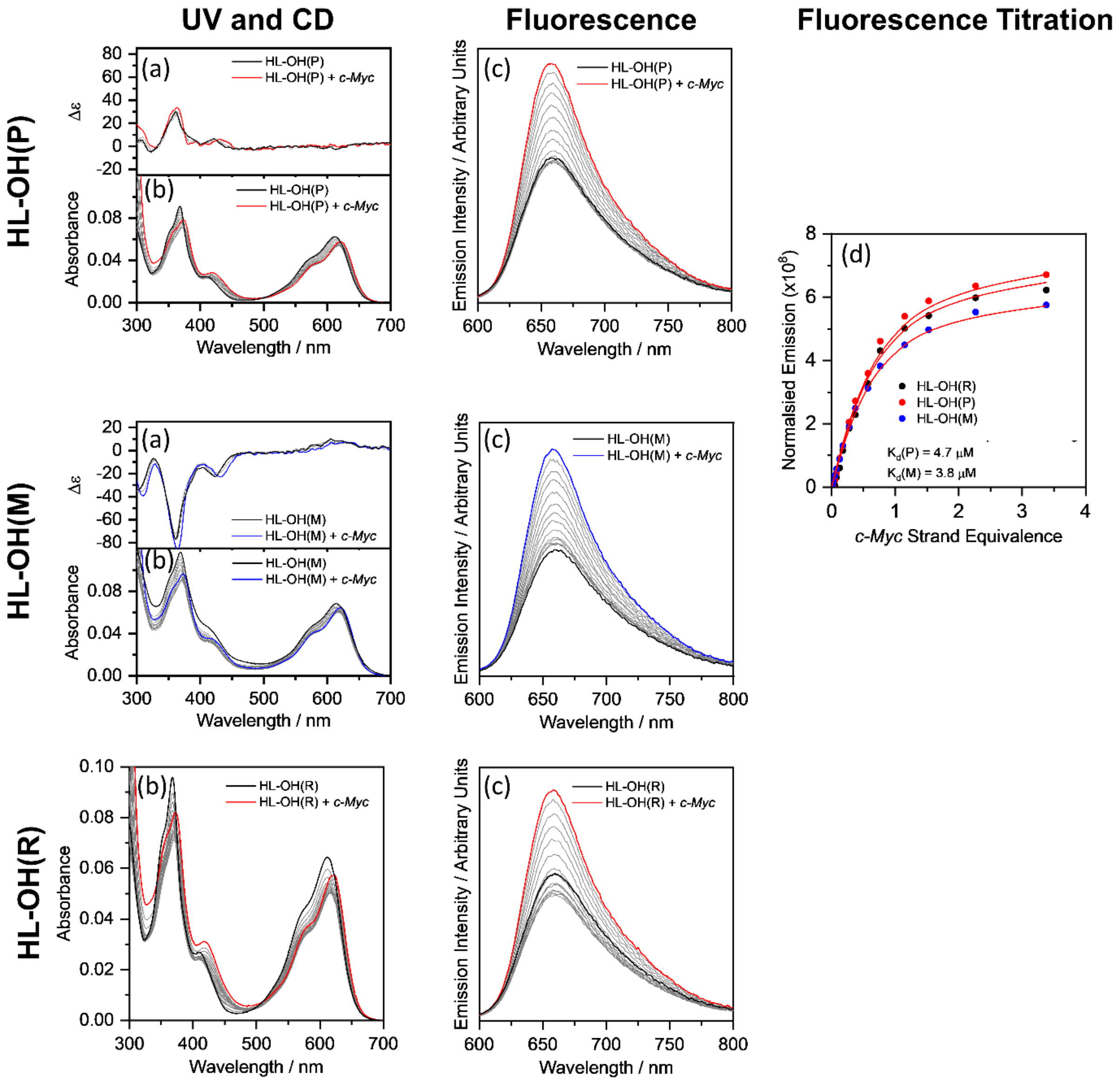

Figure S15. Titration spectra and competitive binding affinities of **HL-OH(R)** (6 µM, 0% ee), **HL-OH(P)** (6 µM, 32% ee), and **HL-OH(M)** (6 µM, 96% ee) to *c-Myc.* (a) CD spectra during addition of *c-Myc*. (b) Absorption spectra and (c) Fluorescence spectra (λ_{ex} = 580 nm, λ_{ex} = 600-700 nm) during addition of *c-Myc*. (d) Difference in Integrated emission during titration. Integrated intensities are normalised against absorption at the excitation wavelength. The solid red lines are a simultaneous best fit of all three titrations to a competitive binding model, to solve for K_P, K_M, $k_{\Delta GP}$ and $k_{\Delta GH}$. All experiments in 10 mM lithium cacodylate buffer (pH 7.3) with 100 mM KCl. For titration data for **HL-OH(R)** see Figure S8.

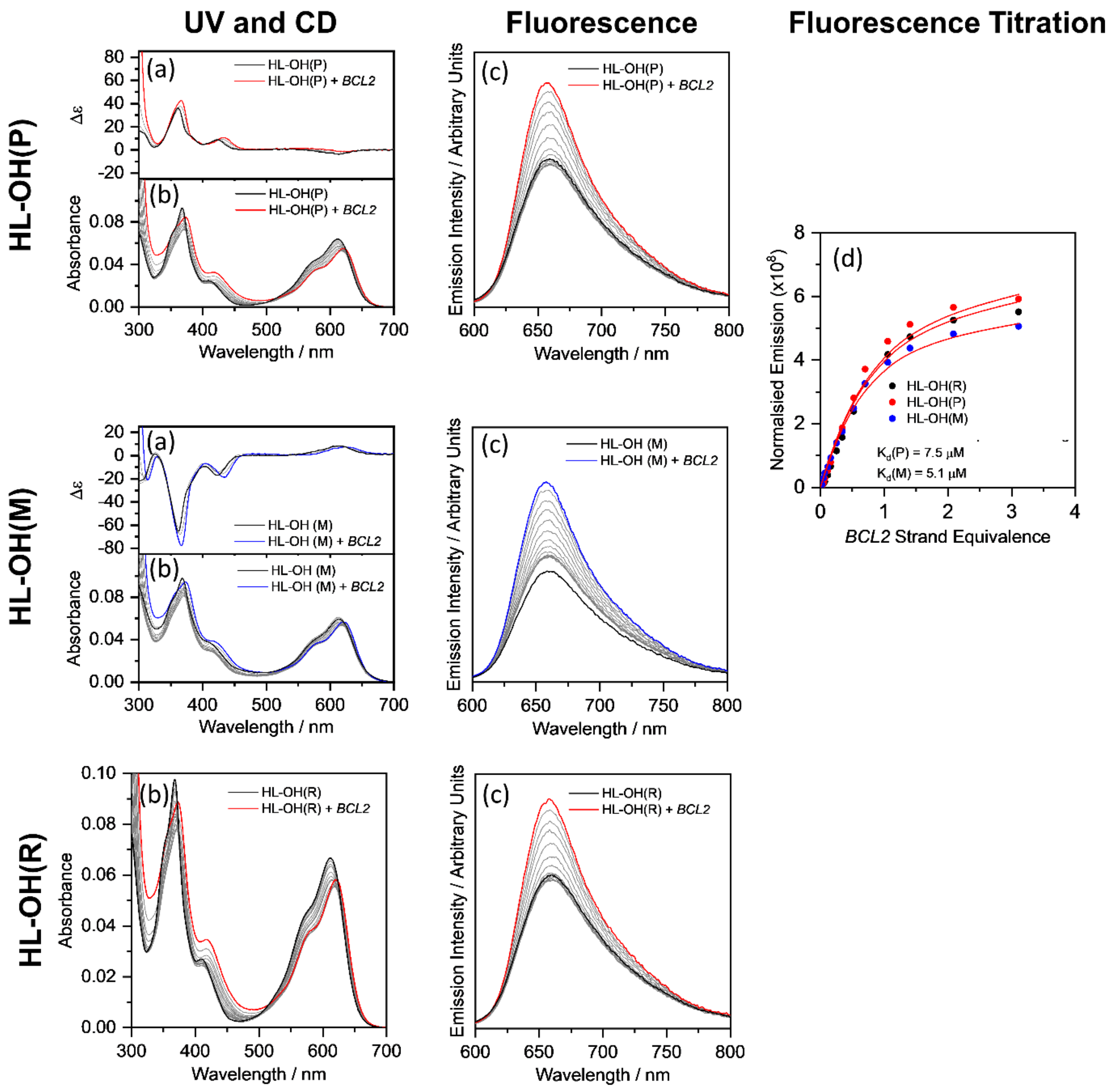

Figure S16. Titration spectra and competitive binding affinities of HL-OH(R) (6 µM, 0% ee), HL-OH(P) (6 µM, 32% ee), and HL-OH(M) (6 µM, 96% ee) to BCL2. (a) CD spectra during addition of BCL2. (b) Absorption spectra and (c) Fluorescence spectra (λex = 580 nm, λex = 600-700 nm) during addition of BCL2. (d) Difference in Integrated emission during titration. Integrated intensities are normalised against absorption at the excitation wavelength. The solid red lines are a simultaneous best fit of all three titrations to a competitive binding model, to solve for KP, KM, kΔGP and kΔGH. All experiments in 10 mM lithium cacodylate buffer (pH 7.3) with 100 mM KCl.

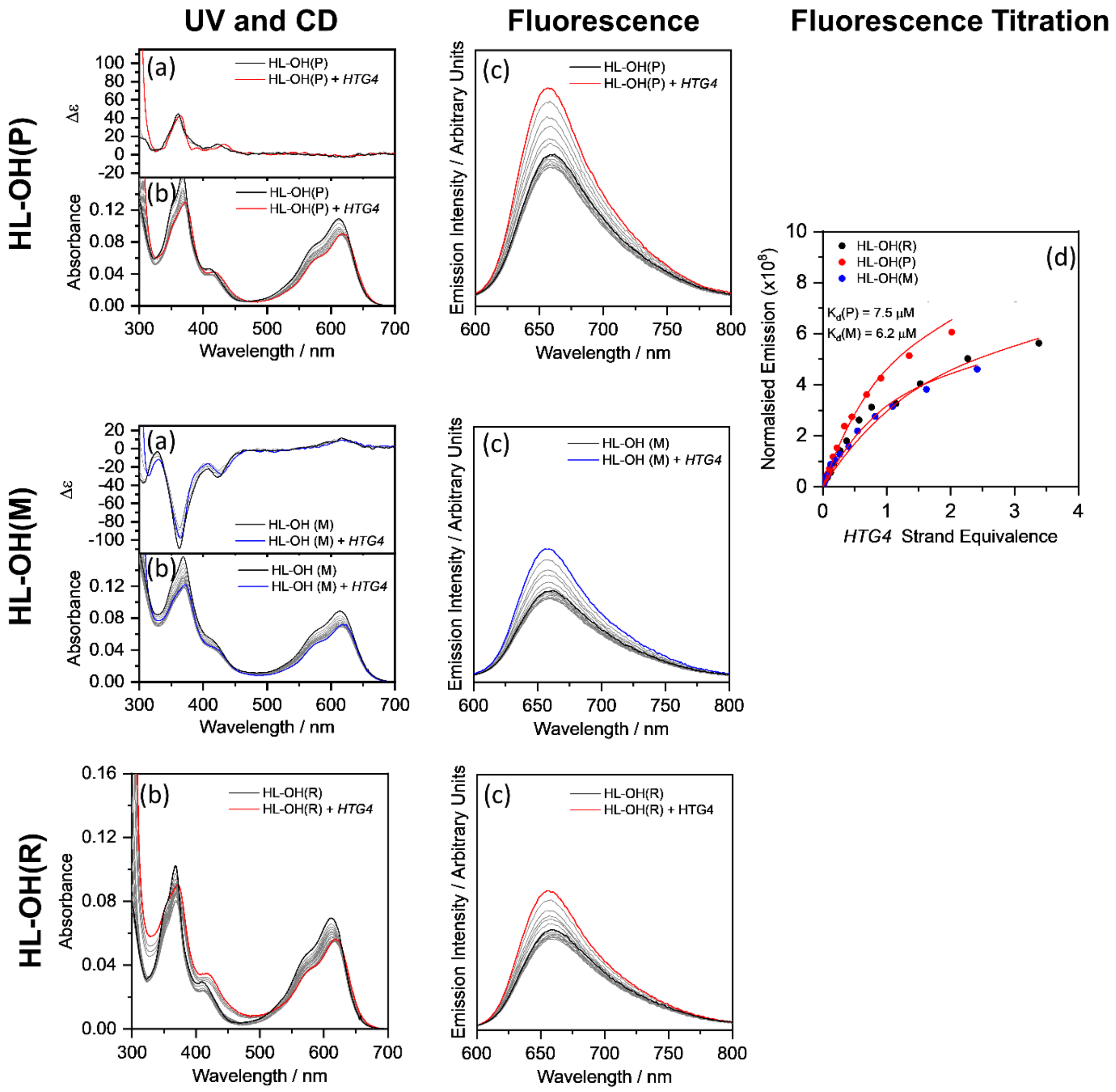

Figure S17. Titration spectra and competitive binding affinities of **HL-OH(R)** (6 µM, 0% ee), **HL-OH(P)** (10 µM, 32% ee), and **HL-OH(M)** (8 µM, 96% ee) to *HTG4.* (a) CD spectra during addition of *HTG4.* (b) Absorption spectra and (c) Fluorescence spectra (λ_{ex} = 580 nm, λ_{ex} = 600-700 nm) during addition of *HTG4*. (d) Difference in Integrated emission during titration. Integrated intensities are normalised against absorption at the excitation wavelength. The solid red lines are a simultaneous best fit of all three titrations to a competitive binding model, to solve for K_P, K_M, $k_{\Delta GP}$ and $k_{\Delta GH}$. All experiments in 10 mM lithium cacodylate buffer (pH 7.3) with 100 mM KCl.

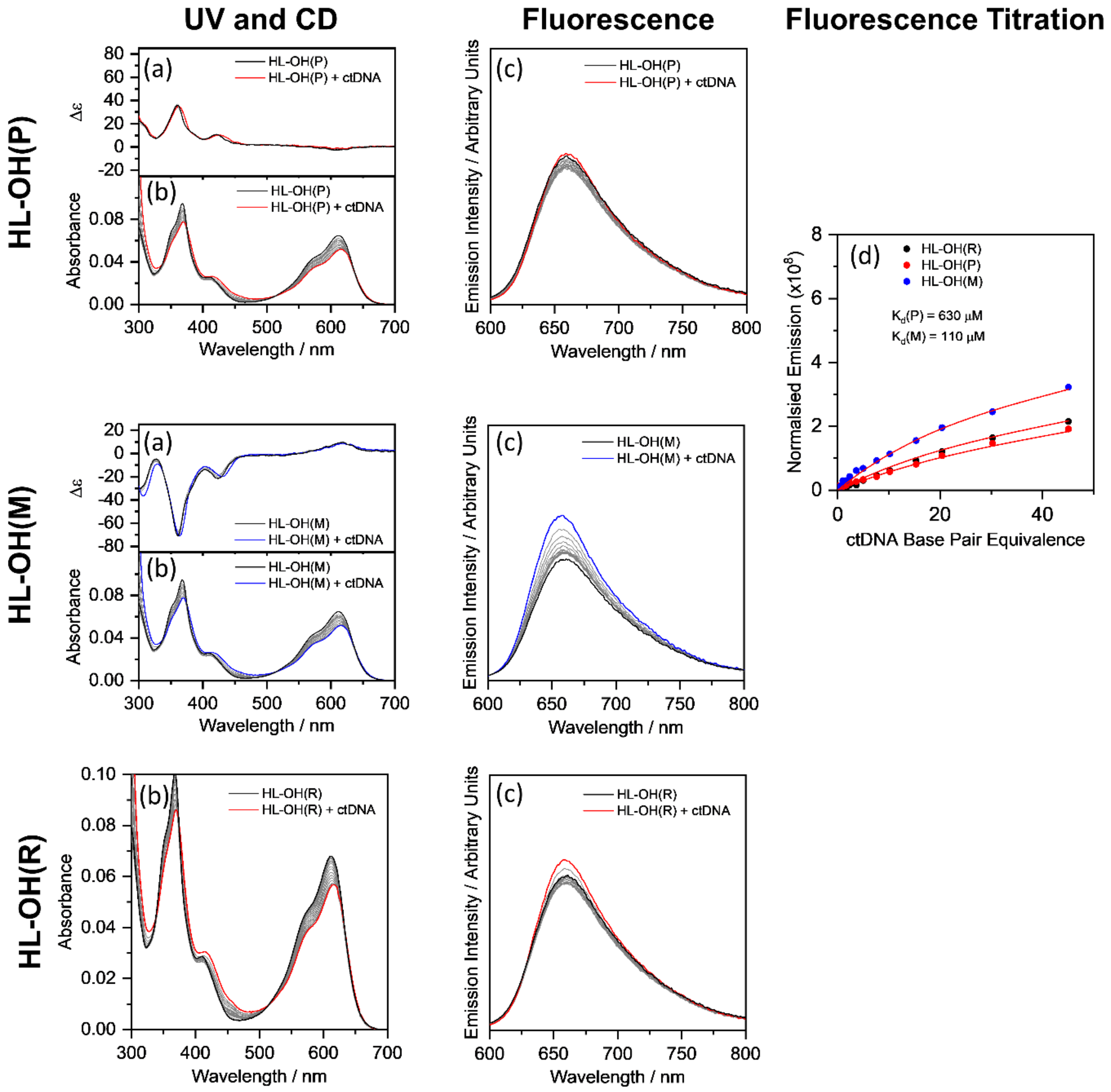

Figure S18. Titration spectra and competitive binding affinities of **HL-OH(R)** (6 µM, 0% ee), **HL-OH(P)** (6 µM, 32% ee), and **HL-OH(M)** (6 µM, 96% ee) to ctDNA. (a) CD spectra during addition of ctDNA. (b) Absorption spectra and (c) Fluorescence spectra (λ_{ex} = 580 nm, λ_{ex} = 600-700 nm) during addition of *c-Myc*. (d) Difference in Integrated emission during titration. Integrated intensities are normalised against absorption at the excitation wavelength. The solid red lines are a simultaneous best fit of all three titrations to a competitive binding model, to solve for K_P, K_M, $k_{\Delta GP}$ and $k_{\Delta GH}$. All experiments in 10 mM lithium cacodylate buffer (pH 7.3) with 100 mM KCl. For titration data for **HL-OH(R)** see Figure S9.

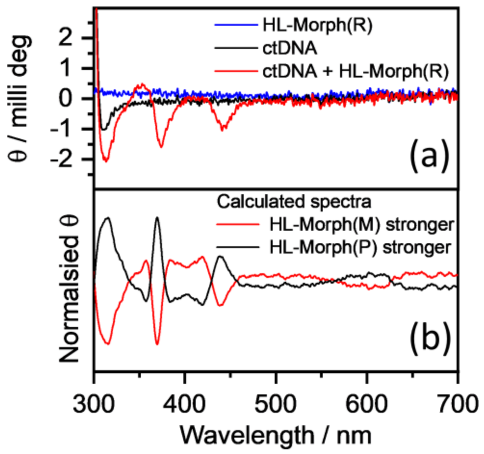

Figure S19. (a) CD Spectra of **HL-Morph(R)** (15 μM, blue line), ctDNA (1.67 mM, black line) and a mixture of **HL-Morph(R)** and ctDNA (10 μM and 1.67 mM, respectively, red line). (b) Calculated spectra assuming either **HL-Morph(M)** or **HL-Morph(P)** is more strongly bound to ctDNA. Calculation based on the CD spectra of **HL-OH(M)** when bound to ctDNA and free in solution [see Figure S18(a)].

As can be seen in Figure S18(a), as ctDNA is added to **HL-OH(M)** the magnitude of the negative CD bands remains almost constant and there is a small red shift. We do not assign this small change in the CD signal to a new chiral arrangement, but rather to a slightly different molecular environment and so altered electronic transition. Essentially, this change is reflected in a small shift in the absorption spectra upon binding to ctDNA [Figure S18(b)].

As it was almost enantiomerically pure (96% ee), we were able to measure the CD spectra of unbound, and ctDNA bound **HL-OH(M)** [Figure S18(a)]. The corresponding CD spectra of **HL-OH(P)** can be estimated from the opposite ellipticity of **HL-OH(M)**. Using these spectra we were able to predict the expected change in the CD signal of **HL-OH(R)** if either the M or the P isomers were to bind to ctDNA more strongly. This result is presented in Figure S19(b). It can be seen that the M isomer binding more strongly clearly recreates the observed spectral pattern more closely than if the P isomer was more strongly bound.

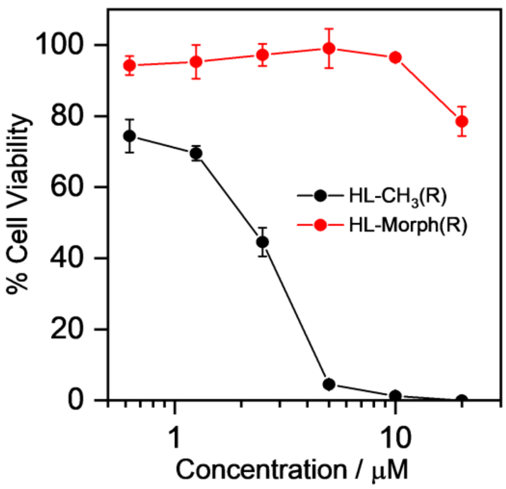

Figure S20. Cytotoxicity of **HL-Morph(R)** and **HL-CH$_3$(R)** towards U2OS cells at 24 hr. Error bars are the ± SD of three measurements.

Additional fluorescence lifetime data:

We have measured the fluorescence lifetimes (τ_W) for enantio-enriched and racemic mixtures of **HL-OH** upon binding to *c-Myc* and *BCL2* G4s (Table S1). While slightly higher lifetimes were detected for the **HL-OH(P)** isomer (which we were not able to purify above 32% ee), compared to **HL-OH(R)** (0% ee) and **HL-OH(P) (**96% ee), we note that there is a more significant variation between lifetimes upon binding to different G4s (*BCL2* > *c-Myc*), as well as the pH dependence of the fluorescence lifetime of all the compounds (Table S2). Thus, we did not examine the lifetime differences for enantiopure/enriched compounds using FLIM. However, we have recorded FLIM of **HL-Morph(R)**, to confirm its binding to DNA *in cellulo*, Figure S21.

Table S1. Fluorescence lifetime (τ_W) of **HL-OH(R)**, **HL-OH(M)**, and **HL-OH(P)** in 10 mM lithium cacodylate buffer (pH 7.3) with 100 mM KCl observed upon binding to excess of G4 DNA.

	Lifetime (τ_W) / ns	
	c-Myc	*BCL2*
**HL-OH(R)** (0% ee)	8.7	9.4
**HL-OH(M)** (96% ee)	8.2	9.2
**HL-OH(P)** (32% ee)	9.1	9.6

Table S2. Fluorescence lifetime (τ_W) of **HL-CH_3(R)**, **HL-Morph(R)**, **HL-OH(R)**, **DAOTA-Morph** and **DAOTA-CH_3** and pH 7.3 and pH 1.0 in 10 mM lithium cacodylate buffer with 100 mM KCl.

	Lifetime (τ_W) / ns	
	pH 1.0	pH 7.3
**HL-CH_3(R)**	5.1	5.1
**HL-Morph(R)**	7.1	2.6
**HL-OH(R)**	2.5	3.8
**DAOTA-Morph**	18.3[a]	2.5[b]
**DAOTA-CH_3**	17.8[c]	17.8[c]

[a] Value from reference 5 in 0.1 M HCl. [b] Value from reference 6. [c] Value from reference 7 in 0.1M HCl.

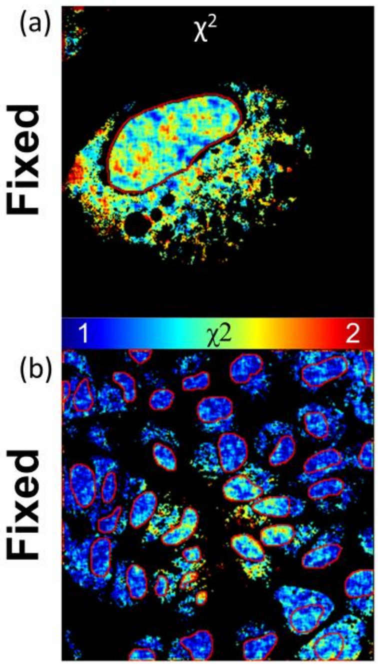

Figure S21 FLIM analysis of fixed U2OS cells stained with **HL-Morph(R)** (20 µM, 0.5 h, λ_{ex} = 640 nm, λ_{ex} = 650-790 nm). χ^2 maps of the FLIM images shown in Figure 4, recorded at (a) 512 x 512 and (b) 256 x 256 resolution.

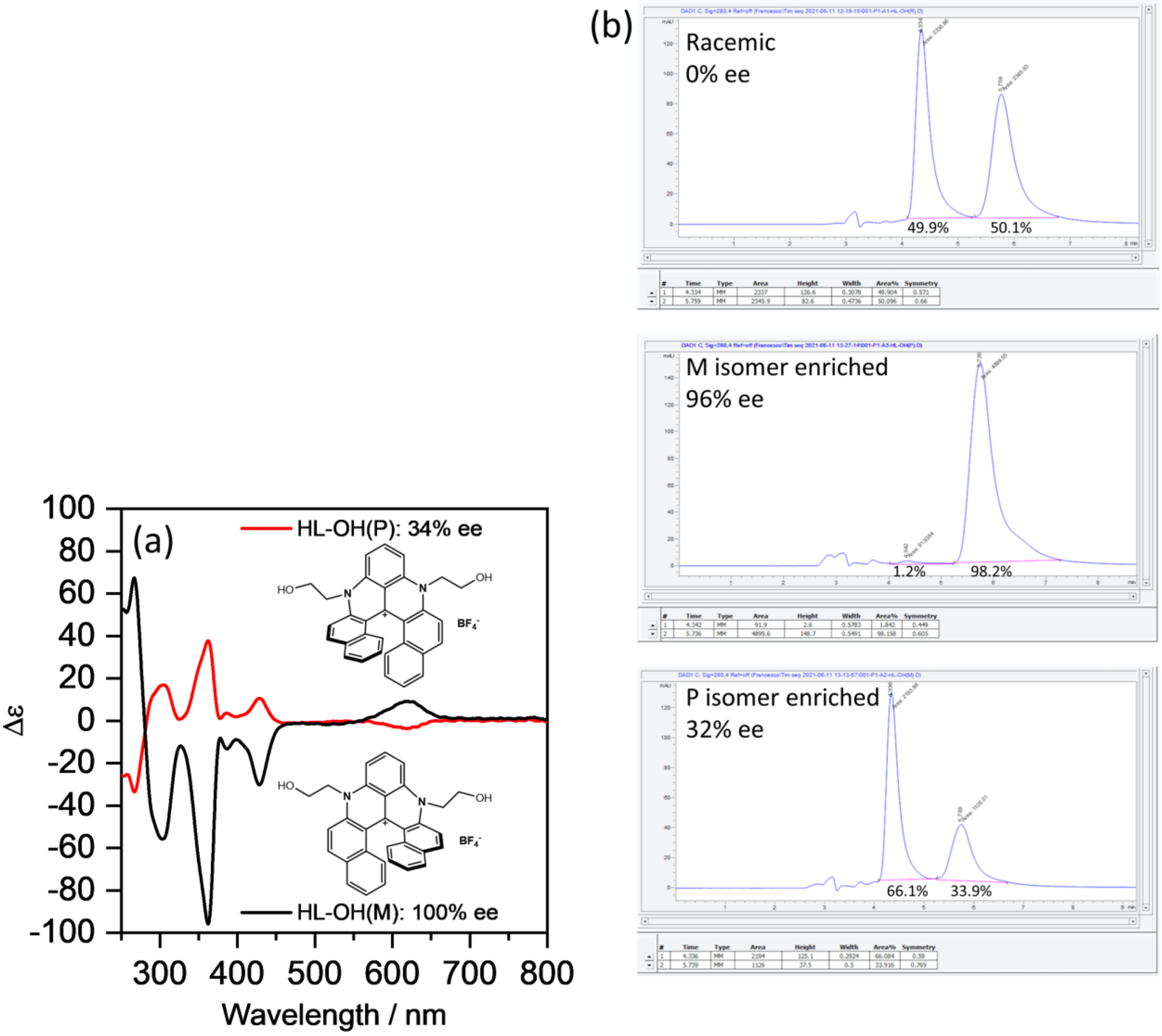

Figure S22. (a) CD Spectra of **HL-OH(P)** and **HL-OH(M)** (12 µM) in CH_2Cl_2. Calculations of enantiomeric excess (ee) are based on the molar circular dichroism (Δε) values for **HL-CH₃(P)** (107 Δε, 96 % ee) and **HL-CH₃(M)** (−87.3 Δε, 92 % ee) in reference 8. (b) Chiral HPLC trace of **HL-OH(R), HL-OH(P)** and **HL-OH(M)** immediately following reduction using $NaBH_4$. Calculations of (ee) are based on the integrated peak areas.

References

1. P. Thordarson, *Chem. Soc. Rev.*, 2011, **40**, 1305-1323.
2. P. Thordarson, *Chem. Soc. Rev.*, 2011, **40**, 5922-5923.
3. I. Jarmoskaite, I. AlSadhan, P. P. Vaidyanathan and D. Herschlag, *eLife*, 2020, **9**, Article e57264.
4. Z. X. Wang, *FEBS Lett.*, 1995, **360**, 111-114.
5. A. Shivalingam, M. A. Izquierdo, A. L. Marois, A. Vyšniauskas, K. Suhling, M. K. Kuimova and R. Vilar, *Nat. Commun.*, 2015, **6**, 8178.
6. P. A. Summers, B. W. Lewis, J. Gonzalez-Garcia, R. M. Porreca, A. H. M. Lim, P. Cadinu, N. Martin-Pintado, D. J. Mann, J. B. Edel, J. B. Vannier, M. K. Kuimova and R. Vilar, *Nat. Commun.*, 2021, **12**, 162.
7. A. Shivalingam, A. Vysniauskas, T. Albrecht, A. J. P. White, M. K. Kuimova and R. Vilar, *Chem. – Eur. J.*, 2016, **22**, 4129-4139.
8. F. Torricelli, J. Bosson, C. Besnard, M. Chekini, T. Burgi and J. Lacour, *Angew. Chem., Int. Ed.*, 2013, **52**, 1796-1800.
